# Roles of fibronectin isoforms in neonatal vascular development and matrix integrity

**DOI:** 10.1371/journal.pbio.2004812

**Published:** 2018-07-23

**Authors:** Heena Kumra, Laetitia Sabatier, Amani Hassan, Takao Sakai, Deane F. Mosher, Jürgen Brinckmann, Dieter P. Reinhardt

**Affiliations:** 1 Faculty of Medicine, Department of Anatomy and Cell Biology, McGill University, Montreal, Quebec, Canada; 2 Department of Molecular and Clinical Pharmacology, Institute of Translational Medicine, University of Liverpool, Liverpool, United Kingdom; 3 Departments of Biomolecular Chemistry and Medicine, University of Wisconsin, Madison, Wisconsin, United States of America; 4 Department of Dermatology and Institute of Virology and Cell Biology, University of Lübeck, Lübeck, Germany; 5 Faculty of Dentistry, McGill University, Montreal, Quebec, Canada; National Institutes of Health, United States of America

## Abstract

Fibronectin (FN) exists in two forms—plasma FN (pFN) and cellular FN (cFN). Although the role of FN in embryonic blood vessel development is well established, its function and the contribution of individual isoforms in early postnatal vascular development are poorly understood. Here, we employed a tamoxifen-dependent cFN inducible knockout (cFN iKO) mouse model to study the consequences of postnatal cFN deletion in smooth muscle cells (SMCs), the major cell type in the vascular wall. Deletion of cFN influences collagen deposition but does not affect life span. Unexpectedly, pFN translocated to the aortic wall in the cFN iKO and in control mice, possibly rescuing the loss of cFN. Postnatal pFN deletion did not show a histological aortic phenotype. Double knockout (dKO) mice lacking both, cFN in SMCs and pFN, resulted in postnatal lethality. These data demonstrate a safeguard role of pFN in vascular stability and the dispensability of the individual FN isoforms in postnatal vascular development. Complete absence of FNs in the dKOs resulted in a disorganized tunica media of the aortic wall. Matrix analysis revealed common and differential roles of the FN isoforms in guiding the assembly/deposition of elastogenic extracellular matrix (ECM) proteins in the aortic wall. In addition, we determined with two cell culture models that that the two FN isoforms acted similarly in supporting matrix formation with a greater contribution from cFN. Together, these data show that pFN exerts a critical role in safeguarding vascular organization and health, and that the two FN isoforms function in an overlapping as well as distinct manner to maintain postnatal vascular matrix integrity.

## Author summary

Fibronectin is a protein that exists in vertebrates in two distinct forms: one present in the blood and the other in blood vessel walls. In mammals, fibronectin is important for the development of blood vessels before birth, but whether it is continuously required for blood vessel homeostasis from birth to adulthood is unknown. We present important results from three genetically modified mouse models, which show that at least one form of fibronectin is required for the proper function and integrity of blood vessels during this period. We show that fibronectin can be transferred from the blood into the vessel wall, where it can rescue the integrity of blood vessels in the absence of the vessel form. This represents an important biological mechanism to maintain the health of blood vessels. Our data also highlight the importance of both fibronectin forms in producing and organizing the microenvironment of cells, with a higher contribution from the fibronectin form residing in the blood vessel walls. Together, our findings show that fibronectin from the blood acts as a safeguard to maintain the health of blood vessels, and both fibronectin forms play crucial roles in development and support of the blood vessel microenvironment.

## Introduction

**Fig 1:**
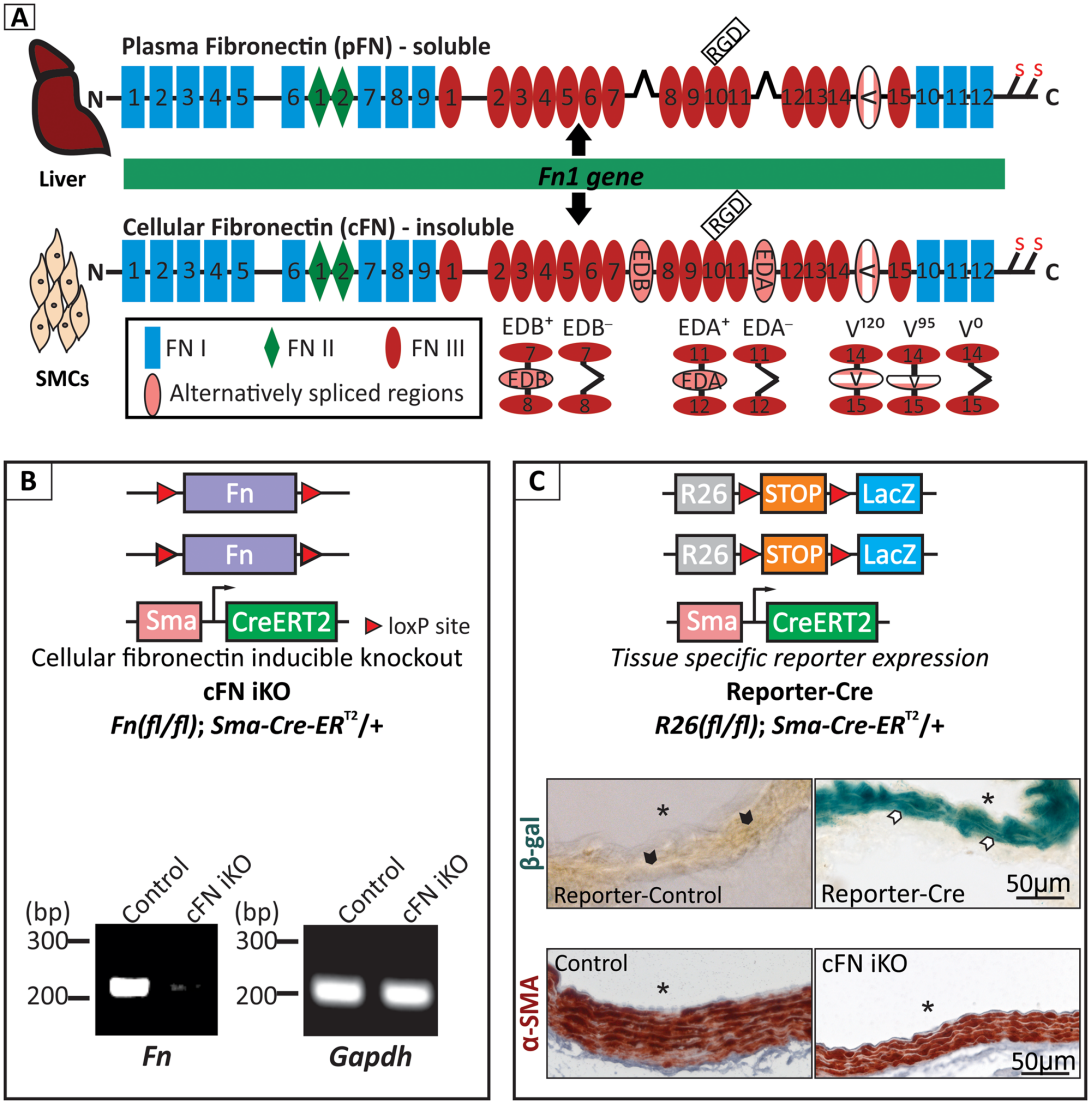
FN and its deletion in the aortic media. **(A)** Schematic diagram of FN isoforms, generated from the single *Fn* gene. pFN is synthesized and secreted by hepatocytes in the liver, and cFN is synthesized by vascular SMCs (vSMCs), among other mesenchymal cells. Alternatively, spliced forms in mouse are indicated. **(B)** Genotype of the smooth muscle specific cFN iKO mouse model (top panel). RT-PCR of *Fn* from total RNA isolated from the aortic media layer, confirming deletion of *Fn* in the cFN iKO post–tamoxifen injection (bottom panel). *Gapdh* was used as a control (*n* = 3). **(C)** Genotype of the *LacZ* Reporter-Cre mouse strain (top panel). β-galactosidase (β-gal) staining of aortae from tamoxifen injected Reporter-control (*R26(fl/fl)*) and Reporter-Cre (*R26(fl/fl); Sma-Cre-ER*^*T2*^*/+*) mice (middle panel) (*n* = 3). Note positive blue staining in the Reporter-Cre (white arrowheads) and the absence of staining in the Reporter-control (black arrowheads) aortae. Immunostaining for α-SMA in control and in cFN iKO mice (bottom panel). Note that the expression of α-SMA in the tunica media is at the same topological location as the β-galactosidase staining in the Reporter-Cre, confirming that the induced Cre recombinase is specifically active in aortic SMCs (*n* = 5). Scale bar represents 50 μm and asterisk (*) denotes aortic lumen in (C). cFN, cellular fibronectin; cFN iKO, cellular fibronectin inducible knockout; CreERT2, Cre recombinase fused with the estrogen receptor tamoxifen-binding domain; FN, fibronectin; *Gapdh*, glyceraldehyde 3-phosphate dehydrogenase; pFN, plasma fibronectin; RGD, Arg-Gly-Asp; RT-PCR, reverse transcriptase polymerase chain reaction; *R26(fl/fl)*, Reporter-control; *R26(fl/fl); Sma-Cre-ER*^*T2*^*/+*, Reporter-Cre; SMC, smooth muscle cell; α-SMA, smooth muscle α-actin; β-gal, β-galactosidase.

Fibronectin (FN) is an abundant and ubiquitously expressed protein in the extracellular matrix (ECM) of various connective tissues as well as in blood of vertebrates [1,2]. Even though coded by a single gene [3], it exists in multiple isoforms as a result of alternate splicing [4]. Each monomer of FN consists of three types of repeating units, FNI, FNII, and FNIII domains (Fig 1A) [5]. Alternative splicing occurs within the central array of FNIII repeats, leading to either inclusion or exclusion of the FNIII extra domain-A (EDA) and extra domain-B (EDB) domains (Fig 1A). Another region of alternative splicing occurs towards the C-terminus of this array, the V region [6]. FNs occur in two principal forms, the soluble plasma FN (pFN) circulating in the blood and the cellular FN (cFN), which polymerizes into insoluble fibers in the ECM of tissues, including blood vessels [7]. pFN is synthesized exclusively in the liver by hepatocytes [8,9] and shows a relatively simple splicing pattern lacking the EDA and EDB domains, although forms with and without the V region exist [10]. The soluble pFN circulates in blood at a high concentration of about 0.6 mg/mL in mice and about 0.3 mg/mL in humans [11,12]. The cFN consists of a much larger and more heterogeneous group of isoforms, with either EDA, EDB, or both domains present [4,13].

FN is broadly expressed in embryos and in adult tissue. It generally regulates a wide spectrum of cellular and matrix related functions that play crucial roles during development, including cell adhesion, migration, growth, differentiation, and tissue repair [5,14]. The pivotal role of FN as a master organizer of ECM assembly is well documented by cell culture studies. The list of ECM proteins that depend on FN for incorporation into the ECM is growing and includes collagen I and III [15–19], fibrillin-1 (FBN-1) [20,21], fibulin (FBLN)-1 [22,23], Latent TGF-β Binding Protein (LTBP)-1 [24], LTBP-4 [25,26], fibrinogen [27], thrombospondin-1 [16], and tenascin-C [28]. FN is not only required for the initiation of these ECM assemblies but also for the stabilization of some of them [16,24,29]. However, whether this holds true in vivo is not established.

The functional relevance of FN in critical physiological and pathological developmental processes is validated by severe defects in mice globally lacking the FN gene (*Fn*) [30], including vascular, mesodermal, and neural tube defects leading to death around embryonic day 9.5. Deletion of both EDA and EDB exons (present only in cFN) from *Fn* in mice also results in embryonic lethality with incomplete penetrance, displaying multiple embryonic cardiovascular defects [31]. Although pFN knockout (KO) mice have a normal life expectancy [32], it is a critical player in various pathologies. pFN supports neuronal survival and reduces infarct size following cerebral ischemia [32]. It is also important in thrombosis, regulating thrombus stability and growth [33,34]. pFN worsens the course of atherosclerosis but promotes the formation of protective fibrous cap, which prevents plaque rupture [35]. In humans, dysregulation of FN levels have been identified in various pathologies with cardiovascular manifestations, including atherosclerosis [36], myocardial infarction [37], Sturge-Weber Syndrome [38], and Ehlers-Danlos syndrome Type X [39]. Altered circulating pFN levels have been reported in patients with coronary heart disease [40,41] and ischemic heart disease [42]. These studies highlight the importance of FN during embryonic vascular development and in postnatal diseases associated with the cardiovascular system.

The aim of this study was to elucidate the role of FN isoforms during postnatal vascular development, with a focus on the elastic fiber-rich tunica media. To specifically delete cFN in smooth muscle cells (SMCs), the most prevalent cells in the tunica media, we employed tamoxifen-dependent Cre recombinase under a smooth muscle specific promoter. To discern the role of pFN in vascular development, a liver-specific Cre mouse was used. Our study demonstrates the importance and function of FN isoforms during the development of the aorta in the early postnatal period.

## Results

It is well documented that FN is crucial in the development of blood vessels during embryogenesis [30,43,44]. The vascular wall further undergoes extensive development during the early postnatal period, including an expansion in total wall volume and wall thickness accompanied by a doubling of SMC number and an increase in matrix volume [45]. The role of FNs during this phase is not known, and thus we addressed it in this study with a focus on cellular and matrix integrity of the aortic vascular media.

### Efficacy and specificity of the inducible cellular FN deletion in the aortic media

We have generated a conditional cFN inducible KO (cFN iKO) mouse model that allows postnatal, tamoxifen-inducible deletion of cFN in SMC, the major cell type in the aortic media. Mice containing *Fn* flanked by loxP sites (*Fn(fl/fl)*) were crossed with an established transgenic mouse line (*Sma-Cre-ER*^*T2*^*/+*), in which the expression of the tamoxifen-dependent Cre-ER^T2^ recombinase is under the control of the mouse smooth muscle α-actin (*α-SMA*) promoter. After several breeding rounds, the cFN iKO genotype (*Fn(fl/fl); Sma-Cre-ER*^*T2*^*/+*) was produced (Fig 1B; top panel) [32,46]. The deletion of *Fn*, induced by intragastric tamoxifen injection (every day from P1–P3), was confirmed at P8 by a *Fn*-specific reverse transcriptase polymerase chain reaction (RT-PCR) of RNA isolated from the aortic media of the cFN iKO compared to control mice (Fig 1B; bottom panel). To further validate the efficiency and specificity of Cre recombinase expression in the *Sma-Cre-ER*^*T2*^*/+* mice following early postnatal tamoxifen injection (P1–P3), a Reporter-Cre mouse strain (*R26(fl/fl); Sma-Cre-ER*^*T2*^*/+*) was generated (Fig 1C; top panel). In this mouse, the *lacZ* reporter gene coding for β-galactosidase is only transcribed in cells and tissues where Cre recombinase is expressed. Intense β-galactosidase staining of the aortic media harvested from tamoxifen-induced Reporter-Cre mice, but not of the tamoxifen-induced Cre negative Reporter-Control (*R26(fl/fl)*) mice, confirmed an efficient and specific expression of the Cre recombinase in the tunica media, identified by α-SMA staining (Fig 1C; middle and bottom panel).

### Postnatal deletion of cellular FN in aortic media leads to a mild phenotype

**Fig 2:**
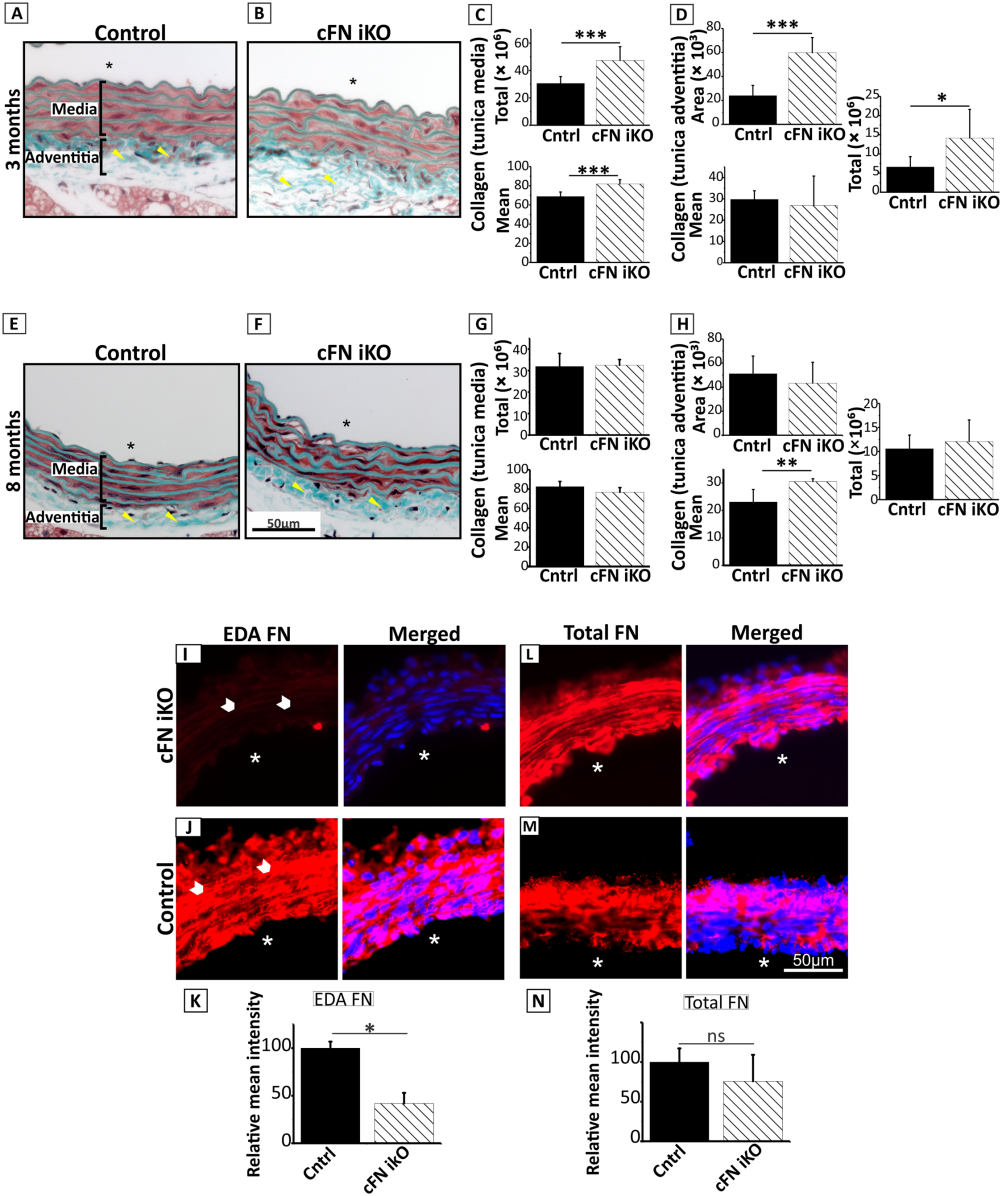
Histological phenotypes in the smooth muscle specific cFN iKO mice. Control mice and cFN iKO mice were treated with tamoxifen from P1 to P3. **(A and B)** Masson’s trichrome stained cross sections of descending aorta at 3 months of age demonstrate higher amounts of collagen deposition in the media and loose collagen deposition in adventitia of cFN iKO mice (turquoise stain) (B) as compared to controls (A) (*n* = 3). **(C and D)** Quantification of collagen in the tunica media (C) and the tunica adventitia **(D)** of picrosirius red stained aorta sections from 3-month-old cFN iKO and control mice (*n* = 4–8). Underlying data are provided in S1 Data. **(E and F)** Masson’s trichrome stained cross sections of descending aorta demonstrate dense collagen deposition in the adventitia of cFN iKO mice at 8 months **(F; triangles)** as compared to controls **(E; triangles)** (*n* = 3). M and A indicate tunica media and tunica adventitia, respectively, and the scale bar represents 50 μm in A–B and E–F. **(G–H)** Quantification of collagen in the tunica media **(G)** and tunica adventitia **(H)** of picrosirius red stained sections of aorta of 8-month-old cFN iKO and control mice (n = 4–8). Parameters quantified are total collagen intensity (Total), mean collagen pixel intensity (Mean), and area of collagen distribution in μm^2^ (Area) in the tunica media/adventitia. Note that the changes in collagen deposition observed at 3 months **(A–D)** revert back at 8 months **(E–H)**. Underlying data are provided in S1 Data. **(I and J)** Immunofluorescence staining of the aortic wall with anti-EDA FN antibody (specific for cFN) shows an effective deletion and reduction of cFN in cFN iKO mice **(I)** as compared to control mice **(J)** (arrowheads) (*n* = 3). **(K)** Quantification of immunofluorescence staining shown in **(I)** and **(J)**. **(L and M)** Immunofluorescence staining of aortae from control and cFN iKO mice with an antibody against total FN (pFN and cFN). Note there is no reduction of total FN staining in the media of cFN iKO mice **(L)** as compared to the control mice **(M)** (*n* = 4). **(N)** Quantification of immunofluorescence staining shown in **(L)** and **(M)**. Underlying data for (K) and (N) are provided in S1 Data. Scale represents 50 μm in Fig 2I, J, L, M. Lumen is indicated with an asterisk in A–B, E–F, I–J, and L–M. For all statistical analyses, * denotes *p* ≤ 0.05, ** denotes *p* ≤ 0.01, and *** denotes *p* ≤ 0.001. cFN, cellular fibronectin; cFN iKO, cellular fibronectin inducible knockout; Cntrl, control–(*Fn(fl/fl*); EDA, extra domain-A; FN, fibronectin; ns, denotes not significant; pFN, plasma fibronectin.

Unexpectedly, tamoxifen-injected cFN iKO mice demonstrated a normal life span in the absence of any gross phenotype. To evaluate aortic tissue integrity of these mice, we performed histological analyses of the aortic wall (Fig 2A–2H). FN guides assembly of several ECM proteins in cell culture models, but whether FN plays a similar role in vivo and what the contributions of the individual FN isoforms are have not been determined. Therefore, one major focus of this study was to analyze the contributions of FN to ECM assembly in the postnatal mouse aorta. FN acts as a scaffold for the assembly of collagen in cell culture [15–19]. However, deleting *Fn* in the tunica media of the cFN iKO mice led to an unexpected increase of collagen deposition at 3 months of age as compared to the control (Fig 2A–2C). Collagen deposition reverted back to control levels at 8 months of age (Fig 2E–2G). Despite the fact that cFN was deleted only in the tunica media, we observed differences in the adventitial collagen organization based on Masson’s trichrome staining and quantification of picrosirius red staining. Up to 3 months of age, the tamoxifen-treated cFN iKO showed a loose collagen deposition in the adventitia (Fig 2B; triangles and Fig 2D), whereas at 8 months, the pattern reverted to a denser collagen matrix as compared to the control (Fig 2F; triangles and Fig 2H). FN also promotes the assembly of fibrillin-1-containing microfibrils in cell culture [20,21]. Immunostaining of aortic sections for fibrillin-1 showed no changes in the aorta of cFN iKO as compared to the control (S1A and S1B Fig). Next, we investigated if postnatal deletion of cFN affects the formation of elastic lamellae in the aortic wall. The elastic lamellae were relatively normal, although they appeared to have more disruptions than the control mice (S1C and S1D Fig). However, quantification of breaks and forks normalized to the area did not show statistically significant differences (S1E and S1F Fig). Overall, postnatal deletion of cFN in medial SMCs led only to minor changes in the matrix organization that did not affect the overall health of the mice.

### Specific uptake of pFN in the vessel wall

Because FN is crucial for vascular development during embryogenesis [30], a much stronger phenotype was expected for the deletion of FN in the aortic wall during early postnatal development. To exclude the possibility that these minor phenotypes in cFN iKO mice were potentially a consequence of incomplete deletion of cFN in the aortic media layer, we immunostained aortic sections using a cFN-specific antibody that recognizes the alternatively spliced EDA domain, which is absent in pFN. This analysis clearly demonstrated that the EDA-containing cFN levels were very low, as expected, from an effective *Fn* deletion (see Fig 1B bottom panel) for the cFN iKO, as compared to the controls (Fig 2I–2K). However, staining with an anti-mouse FN antiserum that recognizes total FN (pFN and cFN) surprisingly revealed the presence of FN in the aortic media of the tamoxifen-induced cFN iKO, similar to the control (Fig 2L–2N). These data led to the hypothesis that pFN from blood can transfer to the aortic wall, which in turn rescues a potential severe phenotype upon cFN deletion in the cFN iKO mice.

**Fig 3:**
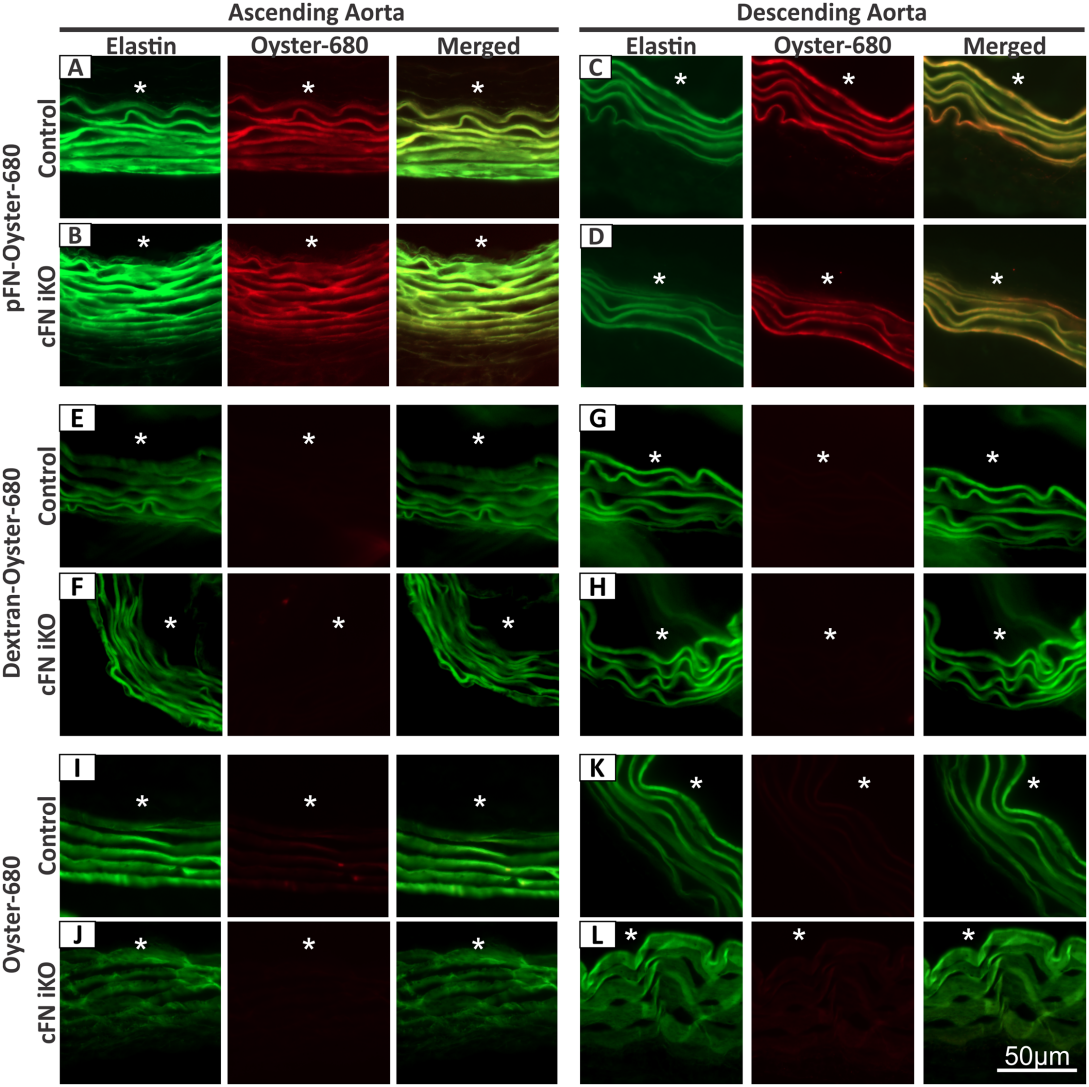
Specific uptake of plasma FN in the aortic vascular wall. pFN or mass-matched dextran was labeled with Oyster-680 (2–3 fluorescent molecules per molecule of pFN or dextran) and injected intraperitoneally from P8 to P10 into tamoxifen-injected (P1–P3) control mice and cFN iKO mice. Oyster-680 alone was used as a control. All mice were dissected on P11. Ascending and descending aortic walls were analyzed by immunofluorescence for elastic lamellae autofluorescence (green signal) and for Oyster-680 fluorescence (red signal). Representative analyses of aortic cross sections from mice injected with fluorescently labeled pFN **(A–D)**, dextran **(E–H)**, and Oyster-680 **(I–L)** are shown. Note that Oyster-680 fluorescence can be detected only in the media of control and cFN iKO mice injected with fluorescent pFN but not in the media of mice injected with fluorescent dextran, or Oyster-680 alone. The Oyster-680–labeled pFN colocalized with the elastic lamellae (see Merged in Fig 3A–D) (*n* = 3–7 for A–L). Lumen is indicated with an asterisk in A–L. cFN iKO, cellular fibronectin inducible knockout; pFN, plasma fibronectin.

To test this hypothesis, exogenous pFN labeled with a fluorophore (Oyster-680), was injected intraperitoneally daily from P8 to P10 into tamoxifen-treated (P1–P3) control and cFN iKO mice, and mice were sacrificed and analyzed at P11. A strong fluorescent red signal in the ascending as well as in the descending aorta of both the control (Fig 3A and 3C) and cFN iKO mice (Fig 3B and 3D) demonstrated an efficient transfer of Oyster-680–labeled pFN into the vessel wall. Within the vessel wall, the Oyster-680–labeled pFN localized to the elastic fibers (merged images in Fig 3A–3D), which were visualized label-free by detection of the elastin autofluorescence. To test if the transfer of intraperitoneally injected pFN to the vessel wall is specific for pFN, an Oyster-680–labeled 250 kDa polysaccharide dextran (similar molecular mass as a pFN monomer) was injected in an identical regimen as used for the fluorophore-labeled pFN. Contrary to the findings with injected pFN, no labeled dextran was observed in the aortic media of ascending and descending aortae of control (Fig 3E and 3G) and cFN iKO (Fig 3F and 3H) mice. As an additional control to exclude the possibility of vascular leakiness, injected Oyster-680 alone did also not transfer into the vessel wall (Fig 3I–3L), despite a much smaller molecular mass (approximately 2 kDa) compared to pFN. These results clearly show that pFN efficiently translocates to the aortic wall in the absence of vascular leakiness.

### Deletion of pFN does not affect the vascular wall

**Fig 4:**
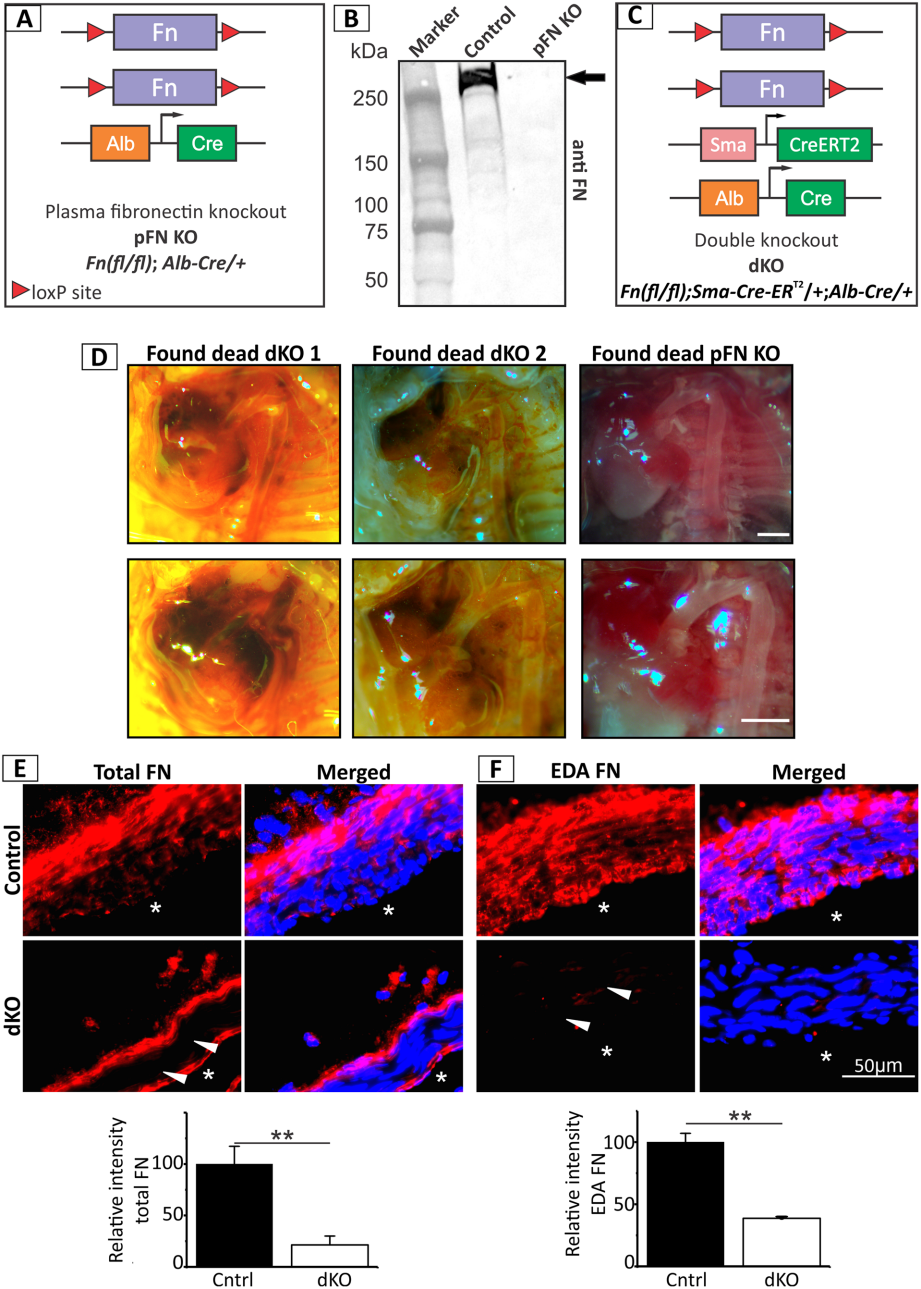
Generation and analysis of pFN KO and dKO mice. **(A)** pFN KO were generated by breeding *Fn(fl/fl)* mice with *Alb-Cre/+* transgenic mice in several breeding rounds. **(B)** Immunoblot for FN using mouse plasma isolated from control and pFN KO mice confirmed the absence of pFN in pFN KO mice (arrow). All mice were 30 days old at the time of blood collection (*n* = 10). **(C)** Generation of dKO mice by breeding *Fn(fl/fl); Sma-Cre-ER*^*T2*^*/+* with *Fn(fl/fl); Alb-Cre/+* mice. After injection with tamoxifen from P1–P3, the dKO mice lack total pFN and cFN secreted by SMCs. **(D)** Hemorrhages in the thoracic cavity of dead dKO mice (P3 and P5) suggest a vascular cause of lethality (left and middle panel). The age-matched control pFN KO mice (found dead) do not show this phenotype (right panel). Animals of each group are shown in the top panel at 3× and in the bottom panel at 4.5× magnification. Scale bars represent 1 mm. **(E** and **F)** Representative immunostained images of aortic walls from P1–P3 tamoxifen-injected control mice and dKO mice at P8 with antibodies against total FN (pFN and cFN) **(E)** and against EDA FN (specific for cFN) *n* = 4 **(F)**. Blue staining represents nuclei counterstaining with DAPI. Quantification of the red FN signals is shown at the bottom of each figure and the underlying data are provided in S1 Data. Note the significantly reduced levels of both total FN and EDA FN in the media (arrowheads). Lumen is indicated with an asterisk, and scale bar represents 50 μm in (E) and (F). cFN, cellular fibronectin; dKO, double knockout; EDA, extra domain-A; FN, fibronectin; KO, knockout; pFN, plasma fibronectin; SMC, smooth muscle cell.

To assess if pFN then plays a more important role in maintaining the cellular and matrix integrity of the vasculature, we generated pFN KO mice by crossing the *Fn(fl/fl)* mice with transgenic mice expressing the Cre recombinase under the control of the albumin promoter (*Alb-Cre*) active in hepatocytes [32,47], the exclusive site for pFN synthesis (Fig 4A). The *Alb-Cre/+* strain has been used successfully by others to generate liver-specific KO mice [32,47,48]. The resulting *Fn(fl/fl); Alb-Cre/+* strain lacks expression of pFN from P3 onwards [49], and the absence was confirmed by immunoblotting of plasma obtained from pFN KO and control mice at P30 (Fig 4B). No gross or histological phenotypes were observed in the aorta of pFN KO mice that would affect cellular or matrix integrity of the vessel wall (S2 Fig). These results together with the observation of pFN transfer into the vascular wall suggested that pFN has an important role in safeguarding the vasculature in the absence of cFN; however, pFN is not needed for vascular integrity and function in the presence of cFN.

### Double knockout of cFN in SMC and pFN causes postnatal lethality

To further study the role of pFN and its functional interaction with cFN in the vasculature, we produced double knockout (dKO) mice deficient in pFN and cFN with the *Fn(fl/fl); Sma-Cre-ER*^*T2*^*/+; Alb-Cre/+* genotype (Fig 4C). These mice lack pFN from P3 onwards and are additionally inducible by tamoxifen to delete cFN in α-SMA expressing tissues. When both pFN and cFN are deleted in the tamoxifen-injected dKO (P1–P3 tamoxifen injection), the mice die postnatally between P3 and P20 (median age of death, P11.5). The Mendelian ratio was significantly reduced from the expected 25% to 4.23% by P30 (Table 1). Necropsy of dead dKO mice showed hemorrhages in the thoracic cavity (Fig 4D), indicating a severe phenotype in the vasculature due to combined loss of pFN and cFN in SMCs. Absence of any phenotypes in non–tamoxifen-injected *Fn(fl/fl); Sma-Cre-ER*^*T2*^*/+; Alb-Cre/+* mice eliminated the possibility of Cre toxicity. These mice had a normal life span and were used as breeders to maintain the mouse colony. Tamoxifen-injected Cre-negative *Fn(fl/fl)* mice also survived normally, demonstrating the absence of tamoxifen side effects. All other analyses throughout this study were performed on mice dissected immediately after cardiac perfusion to optimally preserve the tissue structure. Immunostaining on dissected dKO aortic wall tissues using a FN antibody that recognizes both isoforms showed a complete absence of pFN and cFN in the media layer, as compared to the control (Fig 4E; triangles). Similarly, immunostaining with an EDA-specific antibody confirmed an effective deletion of cFN in the dKO (Fig 4F; triangles). These results validated that both pFN and cFN are normally present in the aortic wall. The postnatal lethality observed in the dKO suggested that at least one of the two FN isoforms is required for maintaining vessel wall integrity and function.

**Table 1:** Genotype and survival analysis of Fn knockout mice.

Genotype	Control^a^ *Fn(fl/fl)*	dKO^a^ *Fn(fl/fl); Sma-Cre-ER*^*T2*^*/+; Alb-Cre/+*	cFN iKO^a^ *Fn(fl/fl); Sma-Cre-ER*^*T2*^*/+*	pFN KO^a^ *Fn(fl/fl); Alb-Cre/+*
Calculated number and percentage of mice expected at P30	17.75 (25%)	17.75 (25%)	17.75 (25%)	17.75 (25%)
Mice alive at P30 (number and percentage)	17 (23.94%)	3 (4.23%)	18 (25.35%)	15 (21.13%)
*p*-values for the difference observed from Mendelian ratios^b^	*p* > 0.9	*p* < 0.001^c^	*p* > 0.9	*p* > 0.5

^a^All mice (control, dKO, cFN iKO, and pFN KO) were injected with 0.1 mg of tamoxifen/day from P1 to P3.

^b^Statistical analysis using the chi-squared test (see Materials and methods).

^c^Indicates a significant *p*-value.

Total mice analyzed = 71.

Total number of mice found dead before P30 = 18.

Abbreviations: cFN iKO, cellular fibronectin inducible knockout; dKO, double knockout; Fn, fibronectin; pFN KO, plasma fibronectin knockout.

### FN isoforms are important for maintaining cellular and matrix integrity of the vasculature

**Fig 5:**
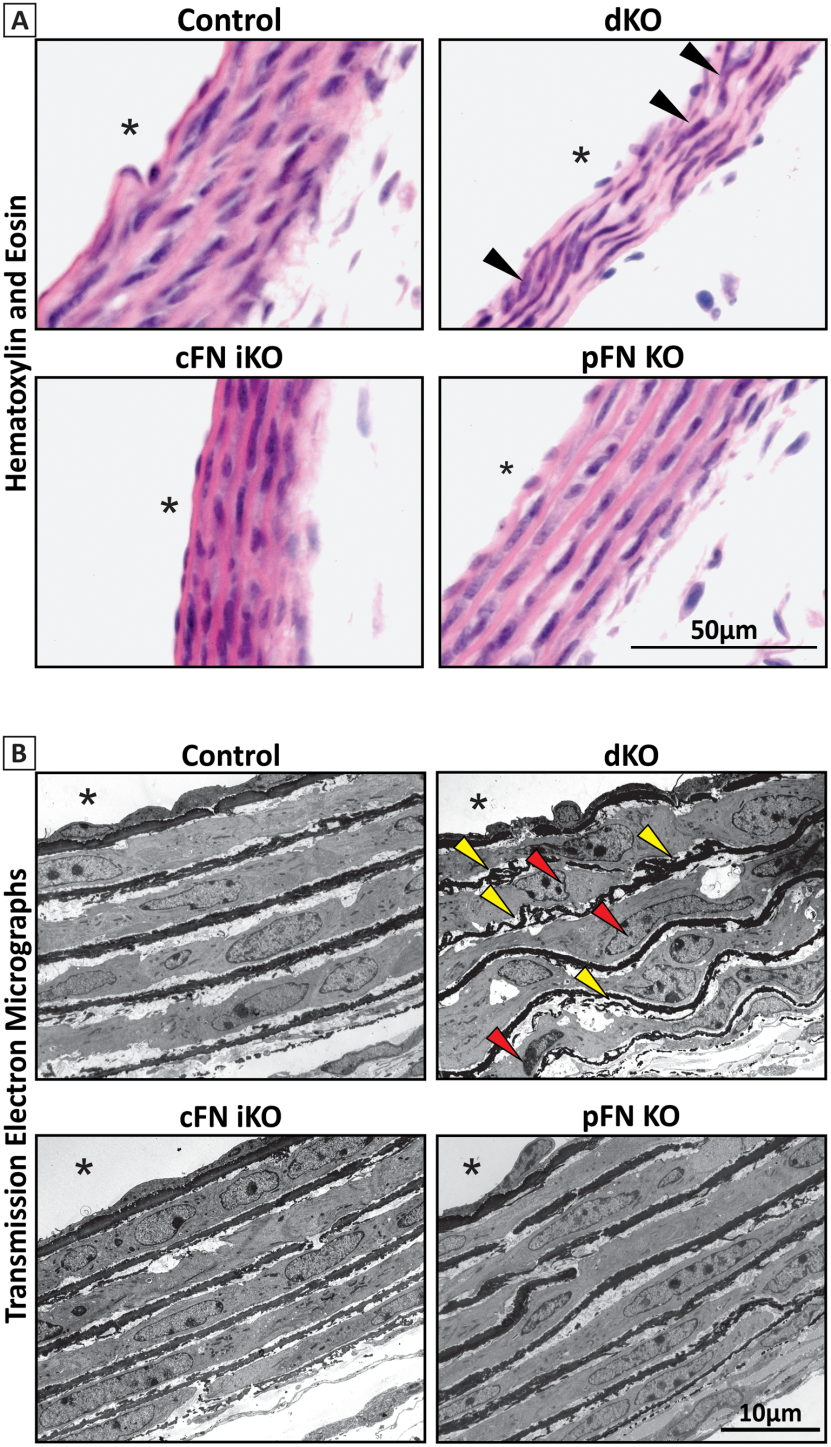
Histological analysis of dKO by bright-field and transmission electron microscopy. All mice were treated with tamoxifen from P1 to P3, and the analysis was performed at P8. **(A)** Hematoxylin and eosin stained cross sections of descending aorta prepared from control, dKO, cFN iKO, and pFN KO mice as indicated. Note the disorganized cells (purple nuclei; black triangles) in the dKO, as compared to the control. **(B)** Transmission electron microscopic analysis of cross sections of descending aorta from control, dKO, cFN iKO, and pFN KO mice. Note that the dKO aorta showed a disorganized tunica media, as compared to the other strains analyzed. Curly disorganized elastic lamellae (yellow triangles) and irregular shaped SMC nuclei (red triangles) are discernible. Scale bar represents 10 μm and asterisk (*) denotes aortic lumen. cFN iKO, cellular fibronectin inducible knockout; dKO, double knockout; pFN KO, plasma fibronectin knockout; SMC, smooth muscle cell.

We further investigated the dKO mice for the consequences of complete FN deficiency on aortic wall development in the early postnatal period. We choose P8 for this analysis, because most dKO mice survive until this time point (median age of death, P11.5). To determine cell integrity, we stained aortic cross sections from all four tamoxifen-injected mouse strains with hematoxylin and eosin. SMCs in the aortic walls from dKO mice appeared to be disorganized, as compared to circumferentially aligned cells in the control aortic sections (Fig 5A; triangles). Frequently, but not always, we observed thinning of the aortic wall in the dKOs, as compared to the other strains analyzed. For a more detailed analysis, we performed transmission electron microscopy on aortic sections. The tunica media of dKO mice appeared disorganized, with curled and irregular elastic lamellae (Fig 5B; S3 Fig; yellow triangles) and irregular shaped cell nuclei (Fig 5B; S3 Fig; red triangles) compared to control and single KO mice.

**Fig 6:**
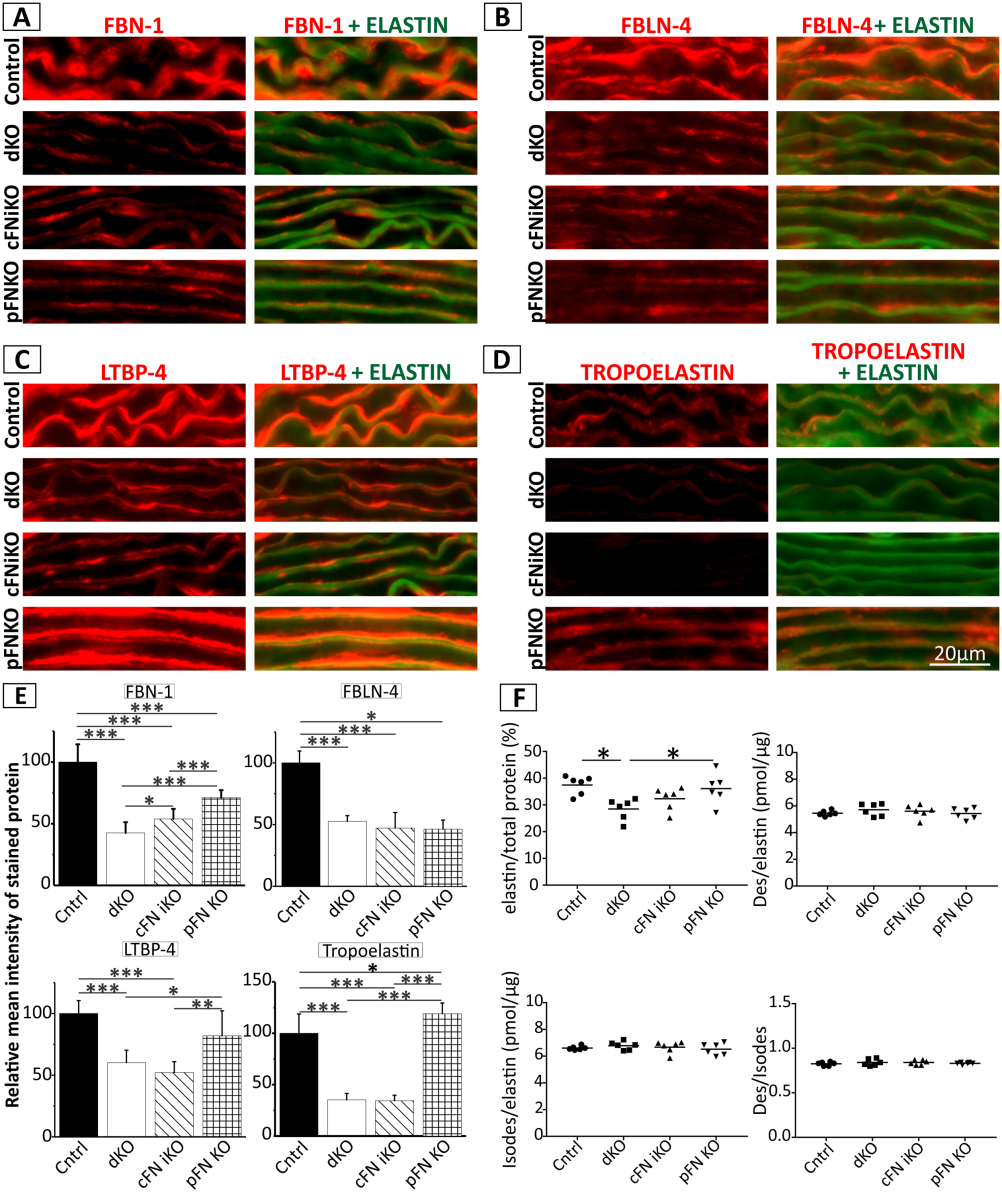
Role of FNs in aortic vessel wall ECM organization. Descending aortic wall sections of tamoxifen-injected control, dKO, single cFN iKO, and single pFN KO mice were analyzed at P8 by indirect immunofluorescence. Shown are representative images stained with antibodies against **(A)** FBN-1, **(B)** FBLN-4, **(C)** LTBP-4, and **(D)** tropoelastin either alone (left panels) or merged with elastic fibers (visualized through autofluorescence) (right panels). **(E)** Quantification of the relative mean intensity of the red signals in immunostained sections shown in A–D. Note that FBN-1 and FBLN-4 are reduced in the dKO as well as in the single cFN iKO and pFN KO, as compared to the control, but only FBN-1 is significantly lower in the dKO, as compared to the single KOs **(A, B, E)**. LTBP-4 and tropoelastin immunostaining is reduced in the dKO and cFN iKO but not in pFN KO **(C, D, E)**. Scale bar represents 20 μm in Fig 6A–D (*n* = 3). Underlying data are provided in S1 Data. **(F)** Biochemical analysis was performed after NaOH extraction of aortae of all four mouse strains dissected at P8 (*n* = 6). Note the decrease in elastin content without changes in elastin cross-links in the dKO. Underlying data are provided in S1 Data. * represents a *p*-value of ≤0.05, ** a *p*-value of ≤0.01 and *** a *p*-value of ≤0.001 in Fig 6E (two-sample *t* test) and F (one-way ANOVA). cFN iKO, cellular fibronectin inducible knockout; Des, desmosine; dKO, double knockout; ECM, extracellular matrix; FBLN, fibulin; FBN-1, fibrillin-1; FN, fibronectin; Isodes, isodesmosine; KO, knockout; LTBP, Latent TGF-β Binding Protein; pFN KO, plasma fibronectin knockout.

The affected elastic lamellae in the dKO led us to further investigate the consequences of *Fn* deletion on matrix integrity of the vascular wall, with a focus on ECM proteins important for elastogenesis. We analyzed FBN-1 in the vessel walls of the KO mice, because it constitutes the backbone of microfibrils [50], which serve as a scaffold for elastin deposition and it is dependent on FN in cell culture studies [20,21]. Immunostaining clearly showed a reduction of FBN-1 in both the single KOs (cFN iKO and the pFN KO) and the dKO, as compared to the control, demonstrating that both pFN and cFN contribute to the deposition/assembly of FBN-1 in vivo (Fig 6A). Quantitative analysis revealed that the levels of deposited/assembled FBN-1 are lowest in the dKO and that cFN supports more FBN-1 deposition/assembly than pFN (Fig 6E). pFN and cFN were similarly important for the deposition/assembly of FBLN-4, albeit it was reduced to the same level in all strains (Fig 6B and 6E). For LTBP-4, which is also known to be dependent on FN for its assembly and is important in elastogenesis [25,51], cFN was important for its deposition/assembly but not pFN (Fig 6C and 6E). To determine if loss of FN affects tropoelastin deposition, either directly or indirectly by affecting other ECM proteins, sections were immunostained with a tropoelastin antibody and mature elastic lamellae were visualized by autofluorescence in aortic sections of all mouse strains analyzed. Even though the mature elastic lamellae appeared relatively normal at this magnification (whereas electron microscopy shows disorganized elastic lamellae in Fig 5B and S3 Fig), immunostaining with tropoelastin antibody was much reduced in sections from cFN iKO and dKO mice, compared to the control and the pFN KO mice (Fig 6D and 6E). Altogether, these data suggest a role of cFN in new tropoelastin deposition and in elastin assembly in vivo. To analyze if this tropoelastin reduction affects the total elastin deposited in the aorta, we biochemically determined the elastin content and cross-links after NaOH extraction of P8 aortae. Surprisingly, the elastin per total protein was significantly reduced in dKO mice, whereas no significant changes occurred in the cFN iKO and pFN KO mice, as compared to the control (Fig 6F). The cross-links desmosine (Des) and isodesmosine (Isodes) per total elastin content and their ratio were not altered between the mouse strains (Fig 6F). mRNA expression analysis of the analyzed ECM components using quantitative polymerase chain reaction (qPCR) demonstrated elevated *Fbn1* mRNA levels in pFN KO aortae, which might represent a mechanism of the tissue to rescue reduced FBN-1 deposition/assembly (S4 Fig). mRNA expression of *Fbln4*, *Ltbp4*, and *Eln* was not altered, demonstrating that FN isoforms exclusively impact the deposition/assembly of these proteins (S4 Fig). In summary, these data show that the complete loss of FN impacts early postnatal vascular development by affecting both cellular and matrix integrity of the vessel wall. The results also demonstrate for the first time that FN is important for the deposition of various elastogenic proteins in vivo and its isoforms have both common and distinct roles in the organization of elastogenic proteins in the aortic media.

### Similar roles of FN isoforms in ECM assembly

**Fig 7:**
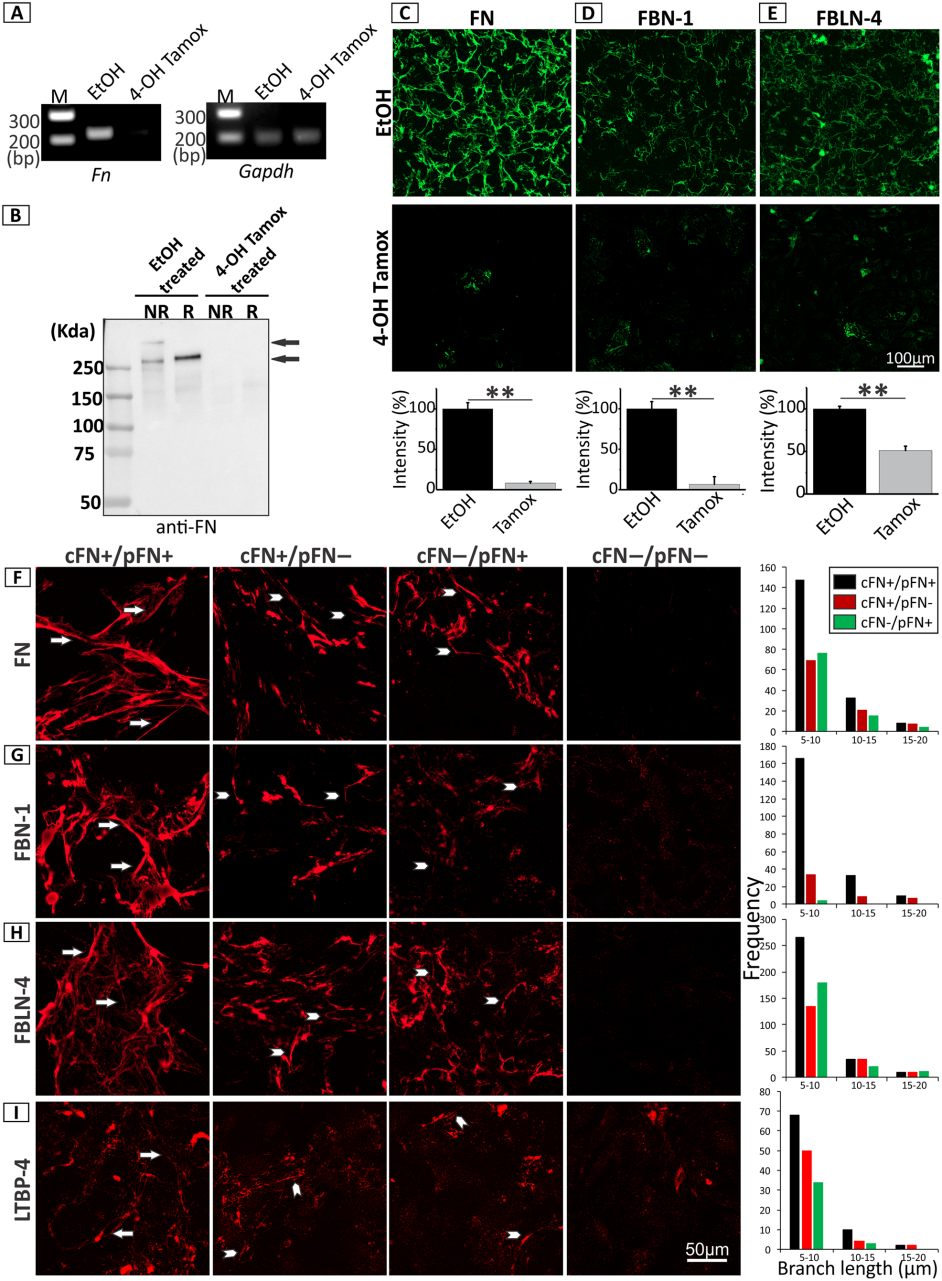
Similar role of FN isoforms in the assembly of elastic fiber related proteins. vSMCs were isolated from *Fn(fl/fl); Sma-Cre-ER*^*T2*^*/+* mouse aortae and grown in medium depleted of pFN. Cells were treated with 4-OH Tamox for 3 days to induce the deletion of cFN, and compared to EtOH-treated controls. **(A)** RT-PCR demonstrates at the mRNA level an effective deletion of *Fn* upon treatment with 4-OH Tamox compared to the EtOH control (219 bp). Gapdh was used as a control (202 bp) (*n* = 3). **(B)** Immunoblot of FN using conditioned cell culture medium (24 hours) showed effective FN deletion on the protein level when cells were treated with 4-OH Tamox (*n* = 4). R indicates reducing conditions with 20 mM Dithiothreitol and NR represents nonreducing conditions. The three arrows indicate monomer, dimer, and higher molecular weight species of FN precipitates from the conditioned medium. **(C-E)** 4-OH Tamox and EtOH treated SMC were stained after 7 days in culture by indirect immunofluorescence against **(C)** FN, **(D)** FBN-1, and **(E)** FBLN-4. Note the absence of FBN-1 and FBLN-4 networks when *FN* is deleted. Scale bar for C–E is 100 μm (*n* = 3). Underlying data are provided in S1 Data. **(F–I)** To test the role of pFN and cFN in facilitating self-assembly and that of FBN-1, FBLN-4, and LTBP-4 in conditions replicating the mouse models, four cell culture conditions were generated (see Materials and methods for details): (1) cFN+/pFN+; (2) cFN+/pFN−; (3) cFN−/pFN+; and (4) cFN−/pFN−. Indirect immunofluorescence analyses were performed after 5 days of cell growth with antibodies against **(F)** FN, **(G)** FBN-1, **(H)** FBLN-4, and **(I)** LTBP-4. Note that in the presence of pFN and cFN individually, short immature fibers develop (arrowheads), whereas the presence of both FN isoforms support enhanced FN self-assembly as well as the assembly and/or deposition of FBN-1, FBLN-4, and LTBP-4 (arrows). The graphs show a histogram distribution of branch lengths for the cFN+/pFN+, cFN+/pFN−, and cFN−/pFN+ samples. No measureable fibers were present in the cFN−/pFN− samples. Underlying data are provided in S1 Data. Total fiber lengths and junction numbers are listed in S1 Table. The scale bar for **F–I** indicates 50 μm (*n* = 4). cFN, cellular fibronectin; EtOH, ethanol; FBLN, fibulin; FBN-1, fibrillin-1; FN, fibronectin; Gapdh, glyceraldehyde 3-phosphate dehydrogenase; LTBP, Latent TGF-β Binding Protein; NR, nonreducing condition; pFN, plasma fibronectin; R, reducing condition; RT-PCR, reverse transcriptase polymerase chain reaction; SMC, smooth muscle cell; 4-OH Tamox, 4-hydroxytamoxifen.

The in vivo data indicated a functional relationship between pFN and cFN in matrix organization. To further study how the two isoforms interact with each other and contribute to its own assembly and deposition of other ECM proteins, a cellular model was developed. vSMCs from *Fn(fl/fl); Sma-Cre-ER*^*T2*^*/+* mouse aortae were treated with 4-hydroxytamoxifen (4-OH Tamox) for 3 days to induce cFN deletion. As controls, the cells were treated with ethanol (EtOH; solvent of 4-OH Tamox). RT-PCR showed an effective deletion of *Fn* upon treatment with 4-OH Tamox as compared to the EtOH-treated control (Fig 7A). Immunoblotting of the serum-free conditioned medium collected from these cells using specific FN antibodies showed an effective reduction of cFN in 4-OH Tamox-treated cells, compared to the EtOH-treated cells (Fig 7B). Indirect immunofluorescence of SMCs cultivated in FN-depleted medium additionally demonstrated the absence of a FN network in 4-OH Tamox-treated cells versus a fully developed network in the EtOH-treated cells (Fig 7C). This result was further confirmed by the absence of FN in deoxycholate (DOC)-soluble and -insoluble fractions extracted from 4-OH Tamox-treated cells (S5 Fig). Deletion of *Fn* prevented the assembly of FBN-1 (Fig 7D) and of FBLN-4 (Fig 7E). This experimental model was then expanded by combining it with plasma obtained from control and pFN KO mice to test the functional relationship between pFN and cFN in matrix organization. The cells were tested under the following conditions: (1) cFN+/pFN+; (2) cFN+/pFN−; (3) cFN−/pFN+; and (4) cFN−/pFN− (for details, see Materials and methods). Indirect immunofluorescence under these conditions and fiber length quantification showed that either cFN or pFN alone (Fig 7F; arrowheads) can form fibers, corroborating the functional in vivo data that showed that either one of the individual FN isoforms is sufficient for maintaining the vascular integrity. FN assembly is also enhanced in the presence of both isoforms (Fig 7F; arrows and S1 Table), suggesting that the two isoforms can assemble together to form matrix fibers. Similarly, each of the two FN isoforms supported the formation of FBN-1 fibers (Fig 7G; arrowheads) as well as the deposition of FBLN-4 (Fig 7H; arrowheads). cFN contributed more than pFN in supporting FBN-1 fibers but equally in FBLN-4 deposition, corresponding with the in vivo data (Fig 6A, 6B and 6E). The results for LTBP-4 (Fig 7I; arrowheads) also correlated with the in vivo role of cFN in promoting LTBP-4 assembly and showed very little contribution of pFN. However, it is important to note that because of the selected sources of FN isoforms (mouse plasma for pFN and SMCs for cFN) to closely mimic the in vivo situation, the actual amounts of individual isoforms could be different. This could contribute to the differences observed between the cFN-containing condition (cFN+/pFN−) and the condition with pFN only (cFN−/pFN+). Tropoelastin and elastic fibers could not be detected in this cell system. For all proteins tested, the presence of both pFN and cFN mediated longer total fiber lengths and more junctions compared to the presence of only one FN isoform, suggesting the two isoforms of FN can contribute together in self-assembly and in guiding the assembly/deposition of other matrix proteins (Fig 7, right panel; S1 Table).

### Distinct roles of FN isoforms in ECM assembly

To analyze if there are actually differences between pFN and cFN in fiber formation and to guide the assembly of other ECM proteins, another cell model was developed that could assess the effects of equal amounts of purified cFN and pFN, unlike the SMC cell culture model, in which cFN was produced by the cells and pFN was present in the mouse plasma added.

**Fig 8:**
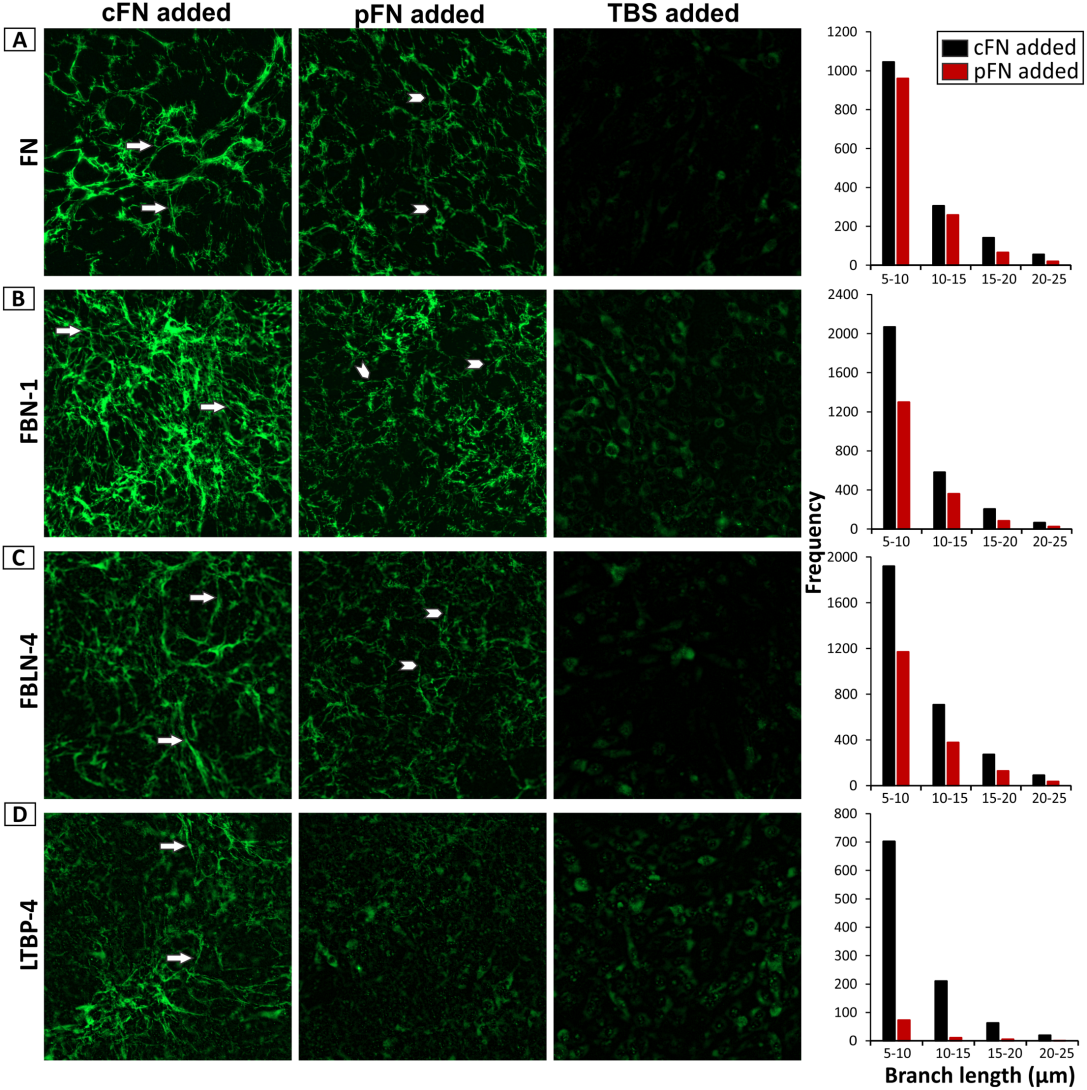
Distinct roles of FN isoforms in guiding ECM assembly. Mouse embryonic fibroblasts from *Fn* null embryos were grown in a pFN-depleted cell culture medium. An equal amount of purified human cFN (25 μg/mL) or human pFN (25 μg/mL), or a TBS control was added to the medium at the time of cell seeding. Indirect immunofluorescence was performed after 8 days of cell growth with antibodies against **(A)** FN, **(B)** FBN-1, **(C)** FBLN-4, and **(D)** LTBP-4. Note the differences in the quality and the quantity of the analyzed fiber systems. cFN supports assembly of longer and more mature fibers (arrows), while pFN forms and supports short and thin fibers (arrowheads). The graphs show a histogram distribution of branch lengths for the cFN added and pFN added samples. No measureable fibers were present in the TBS samples. Underlying data are provided in S1 Data. Total fiber lengths and junction numbers are listed in S2 Table. The scale bar indicates 50 μm for all images (*n* = 3). cFN, cellular fibronectin; ECM, extracellular matrix; FBLN, fibulin; FBN-1, fibrillin-1; FN, fibronectin; LTBP, Latent TGF-β Binding Protein; pFN, plasma fibronectin; TBS, Tris-buffered saline.

Mouse embryonic fibroblasts from *Fn* null embryos are well known to assemble exogenously provided FN [16]. In a culture medium devoid of pFN, these cells grow under FN-free conditions. The absence of any FN fibers in this cell system was validated by indirect immunofluorescence (Fig 8A; Tris-buffered saline [TBS] control). An equal amount (25 μg/mL) of soluble purified pFN or cFN was added into the medium and the quality and quantity of fibers were analyzed. The fibers formed were clearly different between the two FN isoforms (Fig 8A). cFN formed more mature and elongated fibers (Fig 8A; arrows), whereas pFN formed fewer fibers and the fibers were shorter in length (Fig 8A; arrowheads). Similar consequences were observed for both FN isoforms in guiding FBN-1 (Fig 8B) and FBLN-4 (Fig 8C). For LTBP-4, cFN supported assembly/deposition, but pFN could barely support any fiber formation, corresponding well with the in vivo data (Fig 8D). For all proteins tested, cFN mediated longer total fiber lengths and more fiber junctions (Fig 8, right panel; S2 Table). These results demonstrate that pFN and cFN assemble differently and translate these differences onto dependent downstream ECM formation and deposition. Taken together, the data from the two cell culture models and the in vivo results suggest that there are some similar functions but other different roles that cFN and pFN play in self-assembling and in supporting the assembly of other ECM proteins.

## Discussion

FN is a critical player in vascular biology, with essential roles in embryonic development, in various cardiovascular diseases, and in pathologies in which vascular development is a key process, such as in tumor progression. However, virtually nothing is known about the role of FN and its isoforms during physiological postnatal vascular development. Here, we report that deficiency of both hepatocyte-derived pFN and SMC-derived cFN arrests early postnatal aortic development, whereas either one FN isoform is sufficient to support vascular maturation at this developmental stage. We have identified a novel function of pFN in which it represents a safeguard in maintaining blood vessel wall integrity by translocating into the vessel wall. This is also the first report to show, in vivo, the role of FN as a “master organizer” for ECM assembly and to establish that both pFN and cFN have critical functions in vascular wall protein assembly/deposition.

From the initial global KO, it became clear that FN has a critical role in the development and homeostasis of the major blood vessels, as the mice die during embryogenesis around E9.5 with a variety of phenotypes, including defects in blood vessel development [30]. Deletion of both alternatively spliced EDA and EDB exons (present only in cFN) from the *Fn* gene led to embryonic lethality by E10.5, displaying also multiple cardiovascular defects, including vascular hemorrhage, failure of remodeling embryonic and yolk sac vasculature, and defective placental angiogenesis [31]. While cFN is essential for cardiovascular development during embryogenesis, we established from our study that postnatal conditional deletion of cFN in SMCs induced at P1–P3 only results in minor aortic phenotypes not affecting the life span of the mice, as pFN is able to rescue the functions. Similarly, postnatal deletion of pFN around P3 in our study did not result in any obvious aortic phenotype, because SMC-derived cFN is sufficient to maintain the vascular integrity. Therefore, both SMC-derived cFN and pFN are individually dispensable for the postnatal development and homeostasis of the thoracic aorta. However, the deletion of both FNs around P3 resulted in the death of most (>80%) dKO mice until P30. These data demonstrate that at least one of the FNs is required for the continued postnatal development and stability of the thoracic aorta and that pFN can compensate for the loss of SMC-derived cFN. However, Murphy and colleagues reported that when FN was deleted globally (absence of both cFN and pFN) in adult mice, no vascular phenotype was observed, except when the mice were challenged by disturbed blood flow in the carotid arteries, resulting in enlargement of the aorta and hemorrhage [52]. The dependency on at least one—SMC-derived cFN or pFN—during early postnatal aortic development is likely due to the requirement of deposition of new ECM as the aorta grows and matures within the first 4 postnatal weeks [53]. It is known that new elastin deposition during this phase occurs at the fenestrations in the elastic lamellae [54]. As the dKO mice die during this expansion phase, it is possible that FNs support a role in the deposition of new elastin at these fenestrations. This assumption is further supported by (i) ultrastructural tissue analysis, which showed irregular elastic lamellae, and (ii) biochemical data that showed reduced newly deposited elastin at P8 in the dKO mice. Taken together, these ECM deficiencies in the aortic media of the dKO mice are likely the cause for the postnatal lethality of these mice.

cFN iKO mice showed similar matrix defects in the tunica media compared to dKO mice at P8 when analyzed by immunofluorescence (Fig 6). However, at later time points (8 months) no deficiencies in matrix components were observed in the cFN iKO, as compared to control mice (S1 Fig). This indicates that it takes likely somewhat longer than P8 (5 days after tamoxifen injection) for pFN to fully rescue the functions of the deleted SMC-derived cFN, so that the cFN iKO mice develop and survive normally. Our cell culture data also support this hypothesis, as pFN can take over the functions of cFN, but only partially, when analyzed at the same time point (Fig 8).

It was shown in cell culture studies that pFN and cFN have similar but not identical cellular and biochemical functions. For example, the FN isoforms were equally effective in promoting cell spreading and mediating cell attachment to collagen and gelatin substrates [55,56]. On the other hand, EDA/EDB containing cFN incorporated more efficiently into existing fibers compared to pFN [57]. We have identified qualitative and quantitative differences in the FN and FN-dependent fiber systems between the two FN isoforms. cFN formed and supported extended fibers, while pFN formed and guided shorter fibers. This is potentially the result of differential cross-linking of the two isoforms by transglutaminases, where cFN forms a very high molecular weight complex but does not form the intermediate multimers observed in pFN cross-linking [58]. In addition, different transglutaminases act on the two FN isoforms [59].

Our in vivo and cell culture data also provide new insights into the functional relationship of pFN and cFN, in which the two isoforms interact to self-assemble and support the deposition of elastogenic ECM proteins. We and others have previously shown in cell culture that the assembly of fibrillins are strictly dependent on FN fibers [20,21]. It is thought that small units of fibrillin-containing microfibrils are transferred to FN fibers for further elongation and maturation [60]. Here, we demonstrate in cell culture and in vivo that both pFN and cFN support the assembly of FBN-1 and/or the deposition of microfibrils onto the elastic laminae, with greater contribution from cFN. We also identified a FN dependency for FBLN-4 assembly/deposition in cell culture and in vivo, which extends the list of FN-depending ECM fiber systems. It is currently not known, however, whether FBLN-4 interacts directly with FN or through other mediators, potentially including FBN-1. Two other critical elastogenic proteins, LTBP-4 and tropoelastin, showed a differential pattern, both requiring cFN for their assembly/deposition in vivo (and in cell culture for LTBP-4), whereas pFN was not required. The common aspect for both proteins is that their assembly is hierarchically dependent on the presence of FBN-1 and microfibrils [26,61] and thus only indirectly on the presence of FN fibers. One interpretation of these data is that cFN has additional functional or stabilizing consequences (not shared by pFN) on microfibrils that enable them to promote LTBP-4 and tropoelastin assembly. The data for FBN-1 and LTBP-4 are in contrast to what was found in tumors in which nearly complete FN deletion did not affect the assembly/deposition of these proteins [62]. However, in that study, FN deletion was induced much later (5–6 weeks) than in our study, followed by tumor analysis at 12–13 weeks. It is possible that the dependency of FBN-1 and LTBP-4 on FN is primarily required in the early postnatal phase when blood vessels still undergo intensive remodeling and growth, or, alternatively, it is possible that this dependency is tissue specific (aorta versus tumor).

The concept of translocation of pFN from blood into tissues is known for several organs, including liver, spleen, brain, and bone [48,63–65]. Here, we provide novel data showing the function of the translocated pFN in the vascular wall of the aorta. Translocation and accumulation of pFN in the tunica media are not required for normal postnatal development and homeostasis of the aorta, evidenced by the absence of a phenotype in the pFN KO. However, in the absence of SMC-derived cFN in the tunica media, the translocated pFN is able to completely rescue a fetal phenotype (obvious in the dKO), leading to the concept of a critical safeguard function of pFN in maintaining vascular stability and health. This may help explain vascular manifestations in a variety of disorders associated with defective cFN expression, including atherosclerosis [36], myocardial infarction [37], Sturge-Weber Syndrome [38], and Ehlers-Danlos syndrome Type X [39] or altered circulating pFN levels, such as coronary heart disease [40,41] and ischemic heart disease [42].

For the translocation of pFN to the vascular wall, we have excluded a passive diffusion through a potentially leaky endothelial cell layer, as both 250 kDa dextran and the 2 kDa fluorophore do not enter the tunica media. Transcytosis, the transport of molecules across cells through coupled endocytosis and exocytosis transport pathways, is a hallmark of endothelial cells that is frequently mediated through caveolae [66]. Active transcytosis of albumin through endothelial cells, for example, has been shown to be dependent on caveolae [67,68]. While it is not known whether FN uses this pathway, FN can be turned over through integrin β1-mediated (e.g., α5β1) endocytosis regulated by caveolin-1 [69,70]. Thus, it is possible that pFN transcytosis follows a similar transport mechanism.

In summary, this study illuminates the critical role of FN isoforms in postnatal vascular development. It reveals a novel function of pFN, in which it transfers to the vessel walls to safeguard the role of cFN. The two isoforms play important differential and similar functions in maintaining the integrity and matrix stability of the vasculature.

## Materials and methods

### Ethics statement

All animal studies strictly followed the guidelines imposed by the Canadian Council on Animal Care and were approved by the McGill University Animal Care Committee (protocol 2010–5893). Mice were humanely killed by an anesthetic overdose using a ketamine/xylazine/acepromazine cocktail followed by cardiac perfusion.

### Mouse models

*Fn(fl/fl)* mice were generated as previously described [32] and were used as controls throughout the study, unless otherwise mentioned. *Sma-Cre-ER*^*T2*^*/+* mice were a generous gift from Drs. Pierre Chambon and Daniel Metzger and generated as described [46]. *Alb-Cre/+* (B6.Cg-Tg(Alb-cre)21Mgn/J; Stock No. 003574) and *Rosa26(fl/fl)* (B6.129S4-Gt(ROSA)26Sortm1Sor/J; Stock No. 003309) mice were purchased from the Jackson Laboratory. cFN iKO mice (*Fn(fl/fl); Sma-Cre-ER*^*T2*^*/+*) were generated by crossing *Fn(fl/fl)* mice with *Sma-Cre-ER*^*T2*^*/+* mice following several breeding rounds. pFN KO (*Fn(fl/fl); Alb-Cre/+*) mice were obtained by crossing *Fn(fl/fl)* with *Alb-Cre/+* mice after several crossings. Generation of double knockout (dKO) (*Fn(fl/fl); Sma-Cre-ER*^*T2*^*/+; Alb-Cre/+*) mice was achieved by breeding *Fn(fl/fl); Sma-Cre-ER*^*T2*^*/+* with *Fn(fl/fl); Alb-Cre/+* mice. Several breeding rounds were used to produce Reporter-Cre mice (*Rosa26(fl/fl); Sma-Cre-ER*^*T2*^*/+*) from *Sma-Cre-ER*^*T2*^*/+* and *Rosa26(fl/fl)* mice.

### Tamoxifen injection

To delete *Fn* (deleted region encompassing start codon, sequence for signal peptide, and exon/intron border of exon 1) in SMCs of cFN iKO and dKO mice, and to induce β-galactosidase expression in the Reporter-Cre mice, 0.1 mg of tamoxifen (Sigma) was injected intragastrically daily from P1 to P3 using a 2 mg/mL tamoxifen solution in sunflower oil [71]. The same tamoxifen treatment regimen was followed for all the mouse strains analyzed with cFN iKO, dKO, or Reporter-Cre (including control *Fn(fl/fl)*, pFN KO *(Fn(fl/fl);Alb-Cre/+)* and Reporter control *(Rosa26(fl/fl)*)).

### Validation of cFN deletion

To validate *Fn* deletion, RT-PCR was performed using total RNA isolated from descending aorta of 8-day-old mice using the Trizol method (Life Technologies). cDNA synthesis was achieved using SuperScript III kit (Invitrogen). A 219 bp *Fn*-specific PCR product was amplified using *Fn*-sense 5′-CTGAACCAGCCTACAGATGAC-3′ and antisense 5′-CATTTTCTCCCTGCCGATCC-3′ primers and compared to a 202 bp *Gapdh*-specific PCR product obtained with *Gapdh*-sense 5′-GTTGCCATCAACGACCCCTTC-3′ and antisense 5′-ACTCCACGACATACTCAGCAC-3′ primers.

### Tissue-staining procedures

For the analysis of Cre recombinase activity, aortae were harvested from tamoxifen-injected Reporter-Cre (*Rosa26(fl/fl); Sma-Cre-ER*^*T2*^*/+*) and Reporter-control (*Rosa26(fl/fl)*) mice and were embedded in OCT (Sakura Finetek). Eight micrometer cryosections were stained using an established β-galactosidase staining protocol [72].

For histological and immunohistological staining procedures, mice were humanely killed by an anesthetic overdose using a ketamine/xylazine/acepromazine cocktail. Mice were then cardiac-perfused with 10% neutral-buffered formalin, and aortae were harvested. After an overnight incubation in 10% neutral buffered formalin, the tissues were dehydrated through an increasing EtOH gradient and embedded in paraffin. Four micrometer sections were deparaffinized in CitriSolv (Decon Labs) and rehydrated with decreasing EtOH concentrations in H_2_O. The sections were stained with Hematoxylin and Eosin (general histology and cell orientation), Masson’s trichrome stain (collagen), picrosirius red (collagen), or Hart’s elastin stain using standard procedures. For immunostaining, the sections were treated for antigen retrieval with 10 mM citric acid, 0.05% Tween 20 (pH 6) at 98 °C, followed by treatment with bacterial type XXIV proteinase (10 μg/mL; Sigma #P8038). The sections were then incubated with primary antibody (monoclonal anti-human SMA Clone 1A4 [Dako #M0851] at a 1:100 dilution and polyclonal anti-mouse fibrillin-1 C-terminal half [generated in the lab] at 1:3,000). The immunostaining procedure was performed using the Dako Envision+ System-HRP (AEC) staining system (Dako #K4004 and #K4008).

For immunofluorescence staining procedures, the aortae were perfused as above but only fixed in formalin for 1–2 hours. The tissues were then cryoprotected by incubating in a 30% sucrose solution and then embedded in OCT. Eight micrometer cryosections were cut and hydrated in TBS, and blocked with 2% delipidized bovine serum albumin. Primary antibodies were monoclonal anti-EDA FN (Abcam# AB6328; 1:100), polyclonal rabbit anti-mouse FN (Millipore #AB2033; 1:500 diluted), polyclonal rabbit anti-human FBLN-4 [73], 1:500 diluted, polyclonal rabbit anti-mouse FBN-1 (generated in the lab; 1:3,000 diluted), polyclonal rabbit anti-mouse LTBP-4 (generated in the lab; 1:500 diluted), and polyclonal rabbit anti-human elastin (Elastin Products Company #PR398; 1:500 diluted). Secondary antibodies were used at 1:200 dilutions and included Cy5 AffiniPure Goat anti-Rabbit IgG (H+L) and Cy5 AffiniPure Goat anti-Mouse IgG (H+L) (Jackson Laboratories). Cell nuclei were counterstained with 4′,6-diamidino-2-phenylindole, dihydrochloride (DAPI) at a concentration of 2.5 μg/mL.

### pFN and dextran translocation experiments

pFN and dextran translocation experiments were performed with litters injected with tamoxifen (P1–P3) to generate cFN iKOs and controls (*Fn(fl/fl)*). Human pFN (Millipore #FC010) or 250 kDa amino dextran (Fina Biosolutions #AD 250 × 100) were covalently coupled to the water-soluble activated fluorophore Oyster-680 N-hydroxysuccinimide ester (Boca Scientific) in 100 mM NaHCO_3_, 500 mM NaCl, pH 8.4. Oyster-680 emits light at 693 nm, which does not overlap with the known autofluorescence of elastic lamellae in the aorta. Unbound fluorophore was removed by dialysis against the coupling buffer, and the degree of labeling was calculated to be 3–4 molecules Oyster-680 per molecule of pFN or dextran. The labeled pFN or dextran was injected in mice intraperitoneally from P8 to P10 (0.13 mg per mouse per day) using an established protocol [48,74]. TBS-deactivated Oyster-680 alone used at the same molar concentration served as control. Eighteen hours after the last injection, the mice were dissected and tissues harvested after being humanely killed following anesthetic overdose with a ketamine/xylazine/acepromazine cocktail. Eight micrometer OCT-embedded cryosections were analyzed with a Cy5 filter set. Elastin autofluorescence was analyzed using GFP filter set to visualize the elastic lamellae label-free.

### Analysis of pFN in blood

Blood was collected by cardiac puncture from pFN KO and control (*Fn(fl/fl)*) mice after they were humanely killed. Plasma preparation was performed using EDTA-coated tubes (Sarstedt). Three microliter aliquots of plasma were analyzed by standard western blotting under reducing conditions. The polyclonal rabbit anti-mouse FN antiserum (Millipore #AB2033) was used 1:500 diluted as primary antibody, with the 1:200 diluted Peroxidase-AffiniPure Goat Anti-Rabbit IgG (H+L) (Jackson Laboratories) as secondary antibody.

### Necropsy

To investigate the cause of lethality in dKO mice, litters injected with tamoxifen (P1–P3) were monitored twice every day. Dead pups were found often for dKOs but occasionally we found dead pups from other tamoxifen-injected strains, which were used as controls for the necropsy. Dead pups were fixed in 10% neutral buffered formalin (overnight) and washed twice in phosphate buffered saline to prevent postmortem changes. Necropsy was performed to visualize gross anatomy of the dead pups, with a focus on the aorta and thoracic cavity. Images were recorded using an AmScope dissection microscope (ZM-1TZ2-FOR-GT-10M) equipped with a digital camera. All other analyses throughout this study were performed on mice dissected immediately after cardiac perfusion to optimally preserve the tissue structure and avoid any postmortem changes.

### Transmission electron microscopy

For transmission electron microscopic analysis, mice were humanely killed by an anesthetic overdose with a ketamine/xylazine/acepromazine cocktail, followed by cardiac perfusion with 3% glutaraldehyde in 0.1 M sodium cacodylate buffer (pH 7.4) (fixative). The aortae were then harvested and incubated in the fixative overnight. This was followed by a postfixation step with 1% osmium tetraoxide + 1.5% aqueous potassium ferrocyanide. Aortae were then dehydrated with an increasing concentration of acetone, followed by embedding in Epon. Ninety nanometer sections were transferred onto 200 mesh copper TEM grids and counterstained stained using 4% aqueous uranyl acetate and Reynold’s lead before imaging with an FEI Tecnai 12 BioTwin 120 kV transmission electron microscope.

### Biochemical protein analysis

Thoracic aortae from all four mouse strains at P8 (post–tamoxifen injection) were extracted by 0.1 N NaOH at 95 °C for 45 minutes. After centrifugation, the pellets containing insoluble elastin were subjected to hydrolysis and amino acid analysis using a three buffer gradient system and post-column ninhydrin derivatization. The content of elastin cross-links (Isodes and Des) were analyzed in an aliquot of the NaOH-insoluble fraction containing elastin after CF-11 preclearance by amino acid analysis (leucine equivalence factors IDES: 3.4; DES: 3.4). The amino acid composition of the NaOH-insoluble protein fraction was typical for pure elastin [75].

### Quantitative PCR

Descending aortae were harvested from tamoxifen-injected control, dKO, cFN iKO, and pFN KO (littermates) at P8. Total RNA was isolated using the Trizol method (Life Technologies). RNA was reverse transcribed using ProtoScript II First Strand cDNA synthesis kit (New England Biolabs). A QuantStudio 5 Real Time PCR System (Applied Biosystems) was used to perform the quantitative PCR using SYBR Select Master Mix (Applied Biosystems). Specific primers were used for all the genes analyzed. *Gapdh* was used as the control gene. Relative quantification of mRNA levels (fold change) was calculated using the 2^−ΔΔCt^ method. Significance was determined using the two-tailed *t* test.

### Analyses of cell cultures

SMCs were isolated from the tunica media of aortae harvested from *Fn(fl/fl); Sma-Cre-ER*^*T2*^*/+* mice using an established explant-based method [76]. Cells were cultured in DMEM/F12 medium (Gibco) supplemented with 10% heat-inactivated fetal bovine serum and 100 μg/mL penicillin/streptomycin and 2 mM glutamine. Cells were treated with 1 μM of activated 4-OH Tamox for 3 days to induce the deletion of *Fn* (deleted region as described above). EtOH, the solvent for 4-OH Tamox, was used as a control at a final concentration of 0.02% (same concentration as used for the 4-OH Tamox treatment). For experiments, cells were grown in medium containing fetal bovine serum depleted of pFN by gelatin sepharose chromatography [21]. Deletion of cFN was analyzed by RT-PCR from mRNA isolated from cells using RNeasy mRNA extraction kit (Qiagen). RT-PCR was performed as described above. Deletion was also analyzed at protein level by western blotting with 15 μL of conditioned medium (obtained after 24 hours) using 1:500 diluted anti FN antibody (Millipore #AB2033). Indirect immunofluorescence was performed after 7 days with 70% methanol/30% acetone fixed and permeabilized cells. Goat serum diluted in phosphate buffered saline (1:10) was used for blocking. Primary antibodies were prepared in blocking buffer and included polyclonal rabbit anti-mouse FN (Millipore #AB2033–1:500 diluted), anti-mouse FBN-1 (1:500 diluted), and anti-human FBLN-4 (1:500 diluted). Alexa Fluor 488 goat anti-rabbit IgG (H+L) (1:200 diluted) were used as secondary antibodies. DOC extraction was performed using standard procedures as described in [77]. DOC-soluble and -insoluble fractions were analyzed by western blotting using 1:500 diluted anti-FN antibody (Millipore #AB2033).

Experiments were designed to mimic the three KO mouse models and the control in cell culture as follows: 4-OH Tamox and EtOH-treated cells were cultured in the presence of 10% mouse plasma prepared from either wild-type or pFN KO mice. The combinations used were: (1) cFN+/pFN+, EtOH-treated cells (cFN present) were grown in medium containing mouse plasma from control mice (pFN present); (2) cFN+/pFN−, EtOH-treated cells (cFN present) were grown in medium containing mouse plasma from pFN KO mice (pFN absent); (3) cFN−/pFN+, 4-OH Tamox-treated cells (cFN absent) were grown in medium containing mouse plasma from control mice (pFN present); and (4) cFN−/pFN−, 4-OH Tamox treated cells (cFN absent) were grown in medium containing mouse plasma from pFN KO mice (pFN absent). After 5 days, cells were fixed with 4% paraformaldehyde and immunofluorescence was performed as described above, additionally with primary LTBP-4 antibody (1:500 diluted) and secondary Cy5 AffiniPure Goat anti-Rabbit IgG (H+L) antibody (Jackson Laboratories, 1:200 diluted). No LTBP-4 fibers were observed in this setup. However, when, instead of using mouse plasma, 40 μg/mL of purified soluble pFN was added to the system, LTBP-4 fibers formed. This could be due to factors present in the mouse plasma, which might affect LTBP-4 assembly.

Mouse embryonic fibroblasts differentiated from *Fn* null mouse embryonic stem cells were prepared as described [78]. As for the SMCs, the *Fn*^−/−^ embryonic fibroblasts (which do not produce cFN) were grown in pFN-depleted cell culture medium, providing an experimental fibroblast system without pFN and cFN. Affinity chromatography on immobilized gelatin was used to purify human pFN from plasma, as described in detail [79], and cFN from human fibroblast-conditioned medium, as previously described [80]. Twelve and a half micrograms of purified pFN or cFN was added to 500 μL of culture medium at the time of cell seeding. Cells were fixed with 4% paraformaldehyde after an 8 day culture period and analyzed by indirect immunofluorescence, as outlined above.

### Light microscopy imaging

An epifluorescence microscope (AxioImager M2; Zeiss) was used to capture all bright field and immunofluorescence images with an AxioCam ICc5 color camera or an Orca Flash 4.0 sCMOS grayscale camera, respectively. Grayscale immunofluorescence images were false colored using the Zen Pro software (Zeiss).

### Quantification procedures

Mean pixel intensity analysis of various fluorescent signals (EDA FN, Total FN, FBN-1, FBLN-4, LTBP-4, Tropoelastin) was performed with 8-bit TIFF images using the ImageJ software [81] on regions of the tunica media, as described by others [52]. The mean pixel intensity of the background was deducted to produce the values used for plotting the graphs. The error bars in the graphs represent standard deviation.

For collagen analysis, picrosirius red stained images of the aorta were obtained through fluorescence microscopy using a Zeiss HQ Texas Red filter set [82]. Mean pixel intensity, total intensity, and area of collagen distribution analysis in the tunica media and tunica adventitia were performed using the ImageJ software, as described above. Error bars represent standard deviation.

Quantification of total fiber lengths and junction number of ECM fibers visualized by immunofluorescence was performed using the ImageJ distribution, Fiji [83,84]. Color images were converted to 8-bit and the threshold was set to best match the fiber appearance in the original image (same threshold for all the images comparing the same protein staining). This was followed by removing the noise using the despeckle feature, followed by skeletonizing the image using “*Plugins—Skeleton—Skeletonize (2D/3D)*” and analyzing the processed image by clicking “*Analyze—Skeleton—Analyze Skeleton (2D/3D)”* [85]. This provides results about the number of junctions and branch lengths. Branches that were less than 5 μm were excluded to avoid small nonfibrous particles, which can influence the total branch length. All the other branches were added up to get total branch length, and junctions were added to get the total number of junctions per condition. A histogram was generated to depict the number of branches in each condition, binned by branch lengths.

### Statistics

Two-sample *t* test was used to determine significance of the difference in protein staining intensities (tissue and cell immunofluorescence) and mRNA levels (qPCR). One-way ANOVA was used to analyze significance for the difference in elastin content and cross-links (biochemical analysis). Chi-squared test was used to determine the significance of the deviation of mouse strain survival from the Mendelian ratio by comparing the number of observed live mice to that of the expected Mendelian ratio. For all statistical analyses, * denotes *p* ≤ 0.05, ** denotes *p* ≤ 0.01, and *** denotes *p* ≤ 0.001.

## Supporting information

S1 Fig Analysis of matrix integrity of the aorta in cFN iKO mice at 8 months of age. **(A–B)** Fibrillin-1 immunostaining of cross sections of descending aorta show no change between cFN iKO and control at 8 months (*n* = 3). **(C–D)** Hart’s elastin stained cross sections of descending aorta of tamoxifen-injected control and cFN iKO at 8 months. **(E–F)** Quantification of breaks (E) and forks (F) in the elastic lamellae normalized to the area (10,000 μm^2^) of the aortic section analyzed in the cFN iKO and control at 8 months (*n* = 8–10). There was a trend towards more elastin fiber breaks and fewer forks in the cFN iKO, but it did not reach statistical significance. Underlying data are provided in S1 Data. Scale represents 50 μm in A–D. Lumen is indicated with an asterisk in A–D. cFN iKO, cellular fibronectin inducible knockout.
(TIF)

S2 Fig Analysis of cellular and matrix integrity of the aorta in pFN KO mice at P30. **(A–B)** Hematoxylin and eosin stained cross sections of descending aorta from pFN KO (B) and control (A) mice at P30. Note no difference was observed in the organization of SMCs in the tunica media. **(C–D)** Masson’s trichrome staining of cross sections of descending aorta from control (C) and pFN KO (D) mice at P30 showed no changes in collagen deposition. **(E–F)** Immunostaining of cross sections of descending aorta from pFN KO (F) at P30 using fibrillin-1 antibody showed no changes in fibrillin-1 deposition, as compared to the control (E). The lumen is indicated with an asterisk in A–F. pFN KO, plasma fibronectin knockout; SMC, smooth muscle cell.
(TIF)

S3 Fig Disorganized tunica intima in the aorta of dKO mice using transmission electron microscopy. More examples of defective elastic lamellae (yellow triangles) and irregular shaped nuclei (red triangles) observed in the cross sections of dKO aorta on analyzing with transmission electron microscopy. Scale bar represents 10 μm and asterisk (*) denotes aortic lumen. dKO, double knockout.
(TIF)

S4 Fig Quantitative PCR analysis of aortae from FN KO mice. Quantitative PCR was performed with total RNA isolated from descending aortae of tamoxifen-injected mice, as indicated, at P8 (*n* = 3). mRNA levels of the proteins analyzed in immunostaining (Fig 6A–6D) were not altered except for FBN-1, validating the role of FN as a master organizer in ECM protein assembly, but not in mRNA expression. Underlying data are provided in S1 Data. ECM, extracellular matrix; FBN-1, fibrillin-1; FN, fibronectin; KO, knockout.
(TIF)

S5 Fig Analysis of DOC-extracted fractions from vSMCs. Immunoblot of FN using DOC-extracted fractions showed complete absence of FN assembly in 4-OH Tamox-treated vSMCs, as compared to the EtOH-treated cells (*n* = 3). R indicates reducing conditions, with 20 mM dithiothreitol, and NR represents nonreducing conditions. The arrow indicates FN monomers. FN, fibronectin; vSMC, vascular smooth muscle cell.
(TIF)

S1 Data (XLSX)

S1 Table (DOCX)

S2 Table (DOCX)

## Abbreviations

4-OH Tamox: 4-hydroxytamoxifen
α-SMA: smooth muscle α-actin
β-gal: β-galactosidase
cFN: cellular fibronectin
cFN iKO: cellular fibronectin inducible knockout
Cntrl: control–(Fn(fl/fl)
Cre-ERT2: Cre recombinase fused with the estrogen receptor tamoxifen-binding domain
Des: desmosine
dKO: double knockout
DOC: deoxycholate
ECM: extracellular matrix
EDA: extra domain-A
EDB: extra domain-B
EtOH: ethanol
FBLN: fibulin
FBN-1: fibrillin-1
FN: fibronectin
*Fn*: FN gene
Gapdh: glyceraldehyde 3-phosphate dehydrogenase
Isodes: isodesmosine
KO: knockout
LTBP: Latent TGF-β Binding Protein
NR: nonreducing condition
ns: denotes not significant
pFN: plasma fibronectin
qPCR: quantitative polymerase chain reaction
R: reducing condition
RGD: Arg-Gly-Asp
RT-PCR: reverse transcriptase polymerase chain reaction
SMC: smooth muscle cell
TBS: Tris-buffered saline
vSMC: vascular smooth muscle cell
