# Supplementary figures and images for: Roles of fibronectin isoforms in neonatal vascular development and matrix integrity
Source: PLoS Biol. 2018 Jul 23;16(7):e2004812. doi: 10.1371/journal.pbio.2004812 (PMC6072322; doi:10.1371/journal.pbio.2004812)

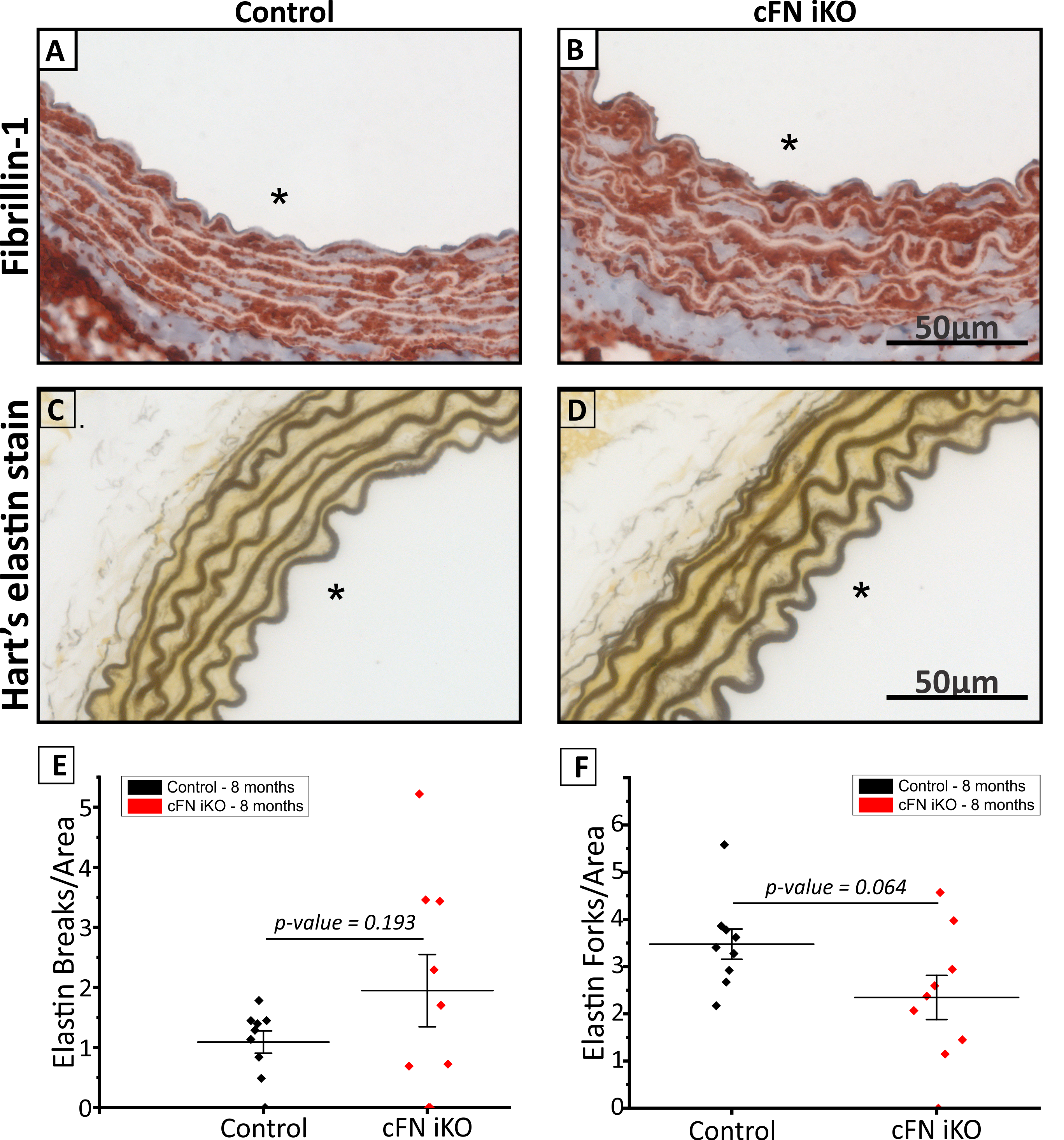

Supplement: S1 Fig — (A–B) Fibrillin-1 immunostaining of cross sections of descending aorta show no change between cFN iKO and control at 8 months (n = 3). (C–D) Hart’s elastin stained cross sections of descending aorta of tamoxifen-injected control and cFN iKO at 8 months. (E–F) Quantification of breaks (E) and forks (F) in the elastic lamellae normalized to the area (10,000 μm2) of the aortic section analyzed in the cFN iKO and control at 8 months (n = 8–10). There was a trend towards more elastin fiber breaks and fewer forks in the cFN iKO, but it did not reach statistical significance. Underlying data are provided in S1 Data. Scale represents 50 μm in A–D. Lumen is indicated with an asterisk in A–D. cFN iKO, cellular fibronectin inducible knockout. (TIF) [file pbio.2004812.s001.tif]

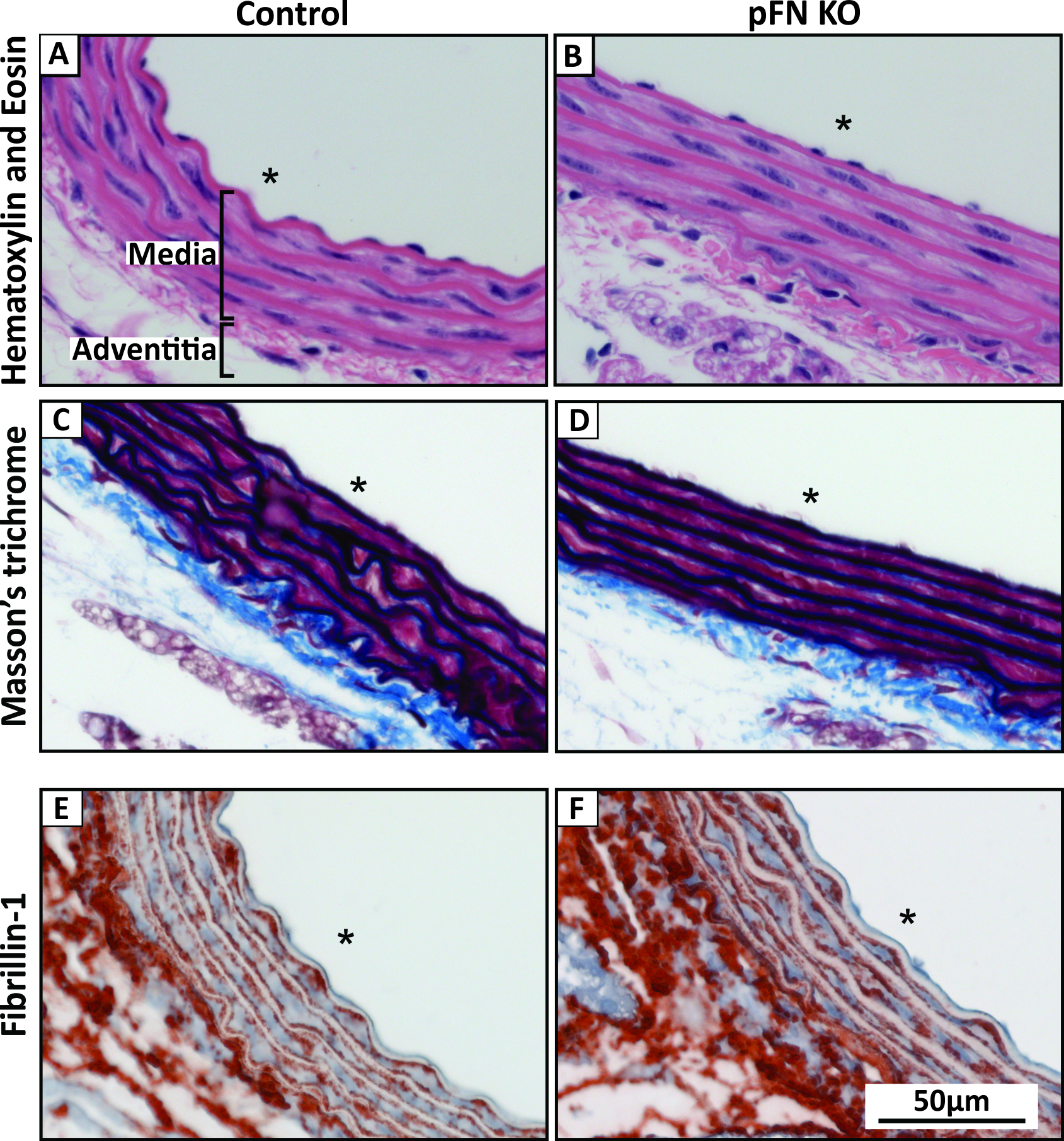

Supplement: S2 Fig — (A–B) Hematoxylin and eosin stained cross sections of descending aorta from pFN KO (B) and control (A) mice at P30. Note no difference was observed in the organization of SMCs in the tunica media. (C–D) Masson’s trichrome staining of cross sections of descending aorta from control (C) and pFN KO (D) mice at P30 showed no changes in collagen deposition. (E–F) Immunostaining of cross sections of descending aorta from pFN KO (F) at P30 using fibrillin-1 antibody showed no changes in fibrillin-1 deposition, as compared to the control (E). The lumen is indicated with an asterisk in A–F. pFN KO, plasma fibronectin knockout; SMC, smooth muscle cell. (TIF) [file pbio.2004812.s002.tif]

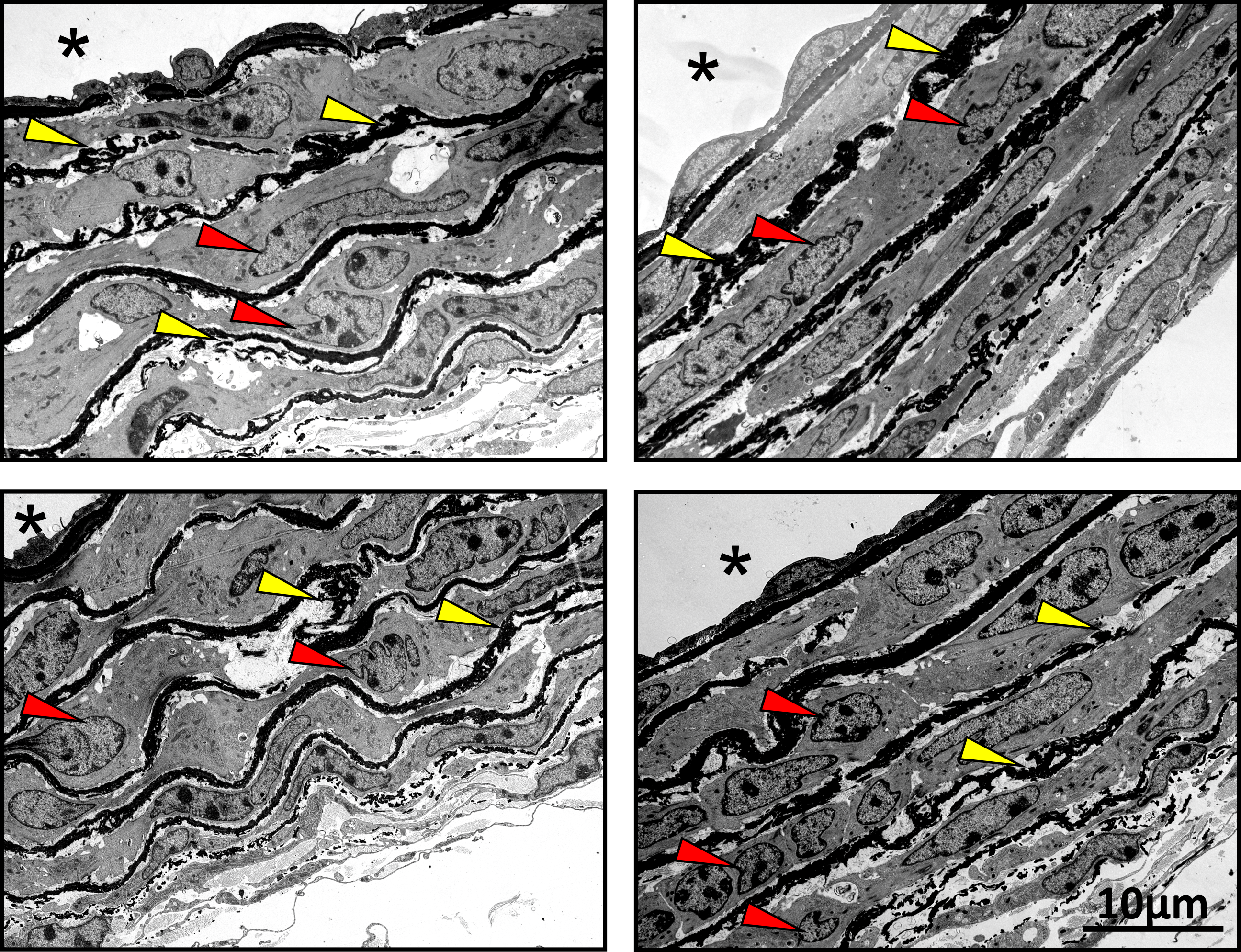

Supplement: S3 Fig — More examples of defective elastic lamellae (yellow triangles) and irregular shaped nuclei (red triangles) observed in the cross sections of dKO aorta on analyzing with transmission electron microscopy. Scale bar represents 10 μm and asterisk (*) denotes aortic lumen. dKO, double knockout. (TIF) [file pbio.2004812.s003.tif]

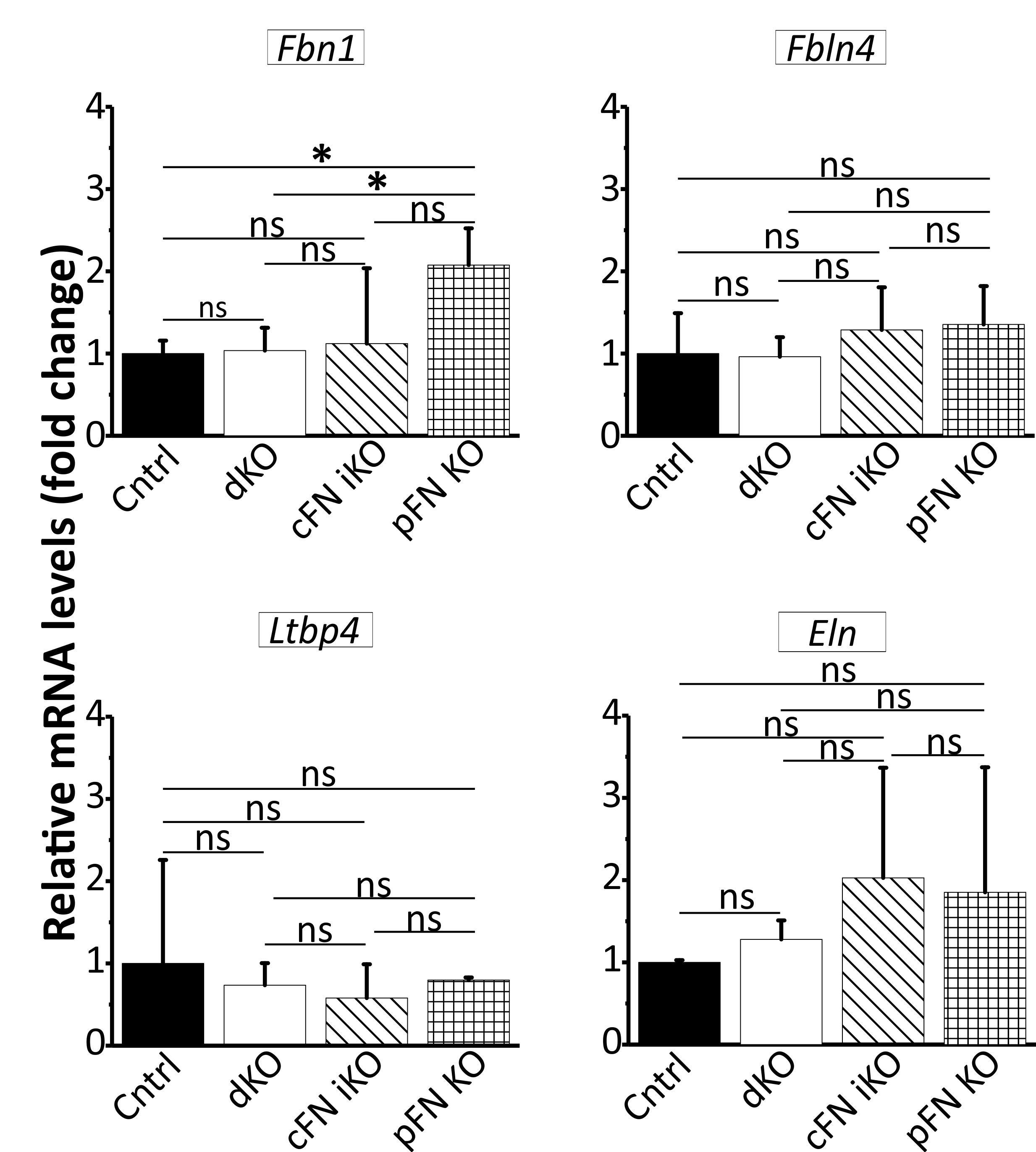

Supplement: S4 Fig — Quantitative PCR was performed with total RNA isolated from descending aortae of tamoxifen-injected mice, as indicated, at P8 (n = 3). mRNA levels of the proteins analyzed in immunostaining (Fig 6A–6D) were not altered except for FBN-1, validating the role of FN as a master organizer in ECM protein assembly, but not in mRNA expression. Underlying data are provided in S1 Data. ECM, extracellular matrix; FBN-1, fibrillin-1; FN, fibronectin; KO, knockout. (TIF) [file pbio.2004812.s004.tif]

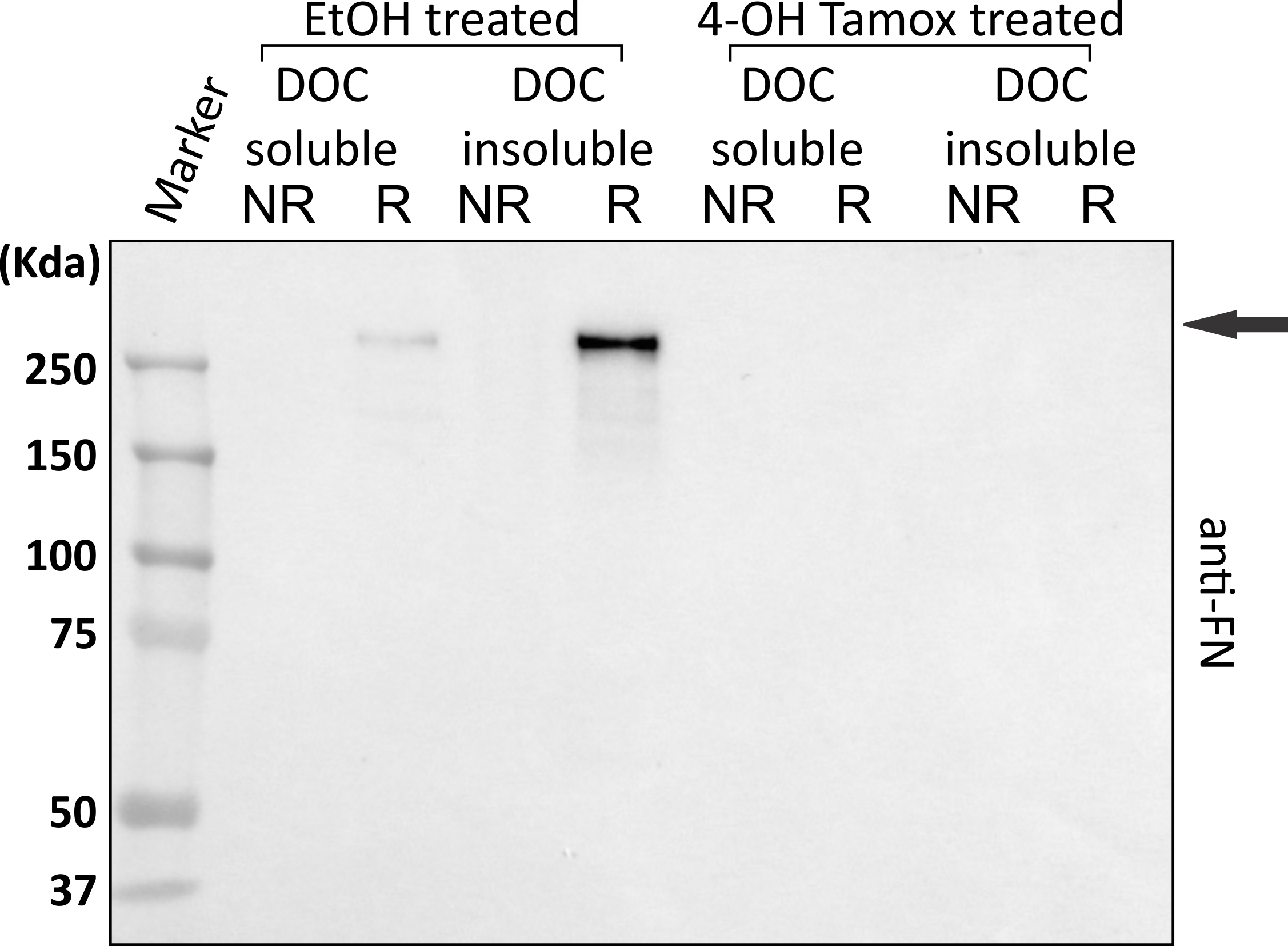

Supplement: S5 Fig — Immunoblot of FN using DOC-extracted fractions showed complete absence of FN assembly in 4-OH Tamox-treated vSMCs, as compared to the EtOH-treated cells (n = 3). R indicates reducing conditions, with 20 mM dithiothreitol, and NR represents nonreducing conditions. The arrow indicates FN monomers. FN, fibronectin; vSMC, vascular smooth muscle cell. (TIF) [file pbio.2004812.s005.tif]
