# Supplementary material for: Roles of fibronectin isoforms in neonatal vascular development and matrix integrity
Source: PLoS Biol. 2018 Jul 23;16(7):e2004812. doi: 10.1371/journal.pbio.2004812 (PMC6072322; doi:10.1371/journal.pbio.2004812)
Supplement: S1 Table — (DOCX) [file pbio.2004812.s007.docx]

**Table S1.** Parameters of extracellular matrix fibers shown in Figure 7F quantified using Fiji (see Materials and Methods).

| **Proteins** | **Parameters** | **cFN+/pFN+** | **cFN+/pFN-** | **cFN-/pFN+** |
| --- | --- | --- | --- | --- |
| **FN** | Fiber length (µM) | 4592 | 2119 | 1969 |
|  | Number of Junctions | 809 | 190 | 343 |
| **FBN-1** | Fiber length (µM) | 4435 | 1245 | 52 |
|  | Number of Junctions | 883 | 164 | 56 |
| **FBLN-4** | Fiber length (µM) | 5468 | 3633 | 3656 |
|  | Number of Junctions | 2583 | 842 | 1387 |
| **LTBP-4** | Fiber length (µM) | 1460 | 903 | 576 |
|  | Number of Junctions | 735 | 589 | 307 |

No measurable fibers present in the “cFN-/pFN-” sample. Numbers are presented per 0.1 mm^2^.
