# Supplementary material for: Roles of fibronectin isoforms in neonatal vascular development and matrix integrity
Source: PLoS Biol. 2018 Jul 23;16(7):e2004812. doi: 10.1371/journal.pbio.2004812 (PMC6072322; doi:10.1371/journal.pbio.2004812)
Supplement: S2 Table — (DOCX) [file pbio.2004812.s008.docx]

**Table S2.** Parameters of extracellular matrix fibers shown in Figure 8 quantified using Fiji (see Materials and Methods).

| **Proteins** | **Parameters** | **cFN added** | **pFN added** |
| --- | --- | --- | --- |
| **FN** | Fiber length (µM) | 3629 | 2692 |
|  | Number of Junctions | 301 | 213 |
| **FBN-1** | Fiber length (µM) | 6251 | 3512 |
|  | Number of Junctions | 598 | 361 |
| **FBLN-4** | Fiber length (µM) | 7070 | 3745 |
|  | Number of Junctions | 554 | 253 |
| **LTBP-4** | Fiber length (µM) | 2107 | 191 |
|  | Number of Junctions | 158 | 8 |

No measurable fibers present in the “TBS added” sample. Numbers are presented per 0.1 mm^2^.
